# Facile Decarbonylation Suppresses the *Ortho* Effect and Ketene Formation in the Catalytic Pyrolysis of Substituted Benzaldehyde Lignin Model Compounds

**DOI:** 10.1021/acs.joc.5c01411

**Published:** 2025-09-25

**Authors:** Xiangkun Wu, Zeyou Pan, Zihao Zhang, Keyong Hou, Saša Bjelić, Andras Bodi, Patrick Hemberger

**Affiliations:** † Environment Research Institute, 12589 Shandong University, Qingdao 266237, China; ‡ 28498 Paul Scherrer Institute, Villigen 5232, Switzerland

## Abstract

Ketene intermediates lead to branching and lower phenol selectivities in the catalytic pyrolysis of lignin model compounds, which makes understanding their formation mechanism key to enable targeted process optimization. While gas-phase pyrolysis of methoxy- and hydroxy-substituted benzaldehydes favors fulvenone ketene formation, it is unclear if the same reaction pathways dominate in the presence of Brønsted acid sites. Thus, we tested if HZSM-5 produces fulvenone utilizing *operando* photoelectron photoion coincidence spectroscopy. Hydroxybenzaldehydes undergo acid-catalyzed decarbonylation, via oxonium mediated hydrogen transfer reactions, to phenol instead of dehydrogenation to fulvenone. The catalytic pyrolysis of anisaldehydes is initiated by demethylation and decarbonylation to yield anisole or hydroxybenzaldehydes and does not produce ketene either. Subsequently, decarbonylation and demethylation, respectively, lead to phenol and methylated derivatives due to abundant surface methyl groups over HZSM-5. Comparative analysis of the catalytic pyrolysis pathways of methoxyphenols and anisaldehydes, reveals that the chemistry of individual functional groups outcompetes the interactions of the vicinal substituents (*ortho* effect) in anis- and salicylaldehydes, resulting in the suppression of fulvenone ketene. We discuss how the high reactivity of aldehyde functionalities by decarbonylation may be leveraged to increase selectivities to value-added products.

## Graphical abstract

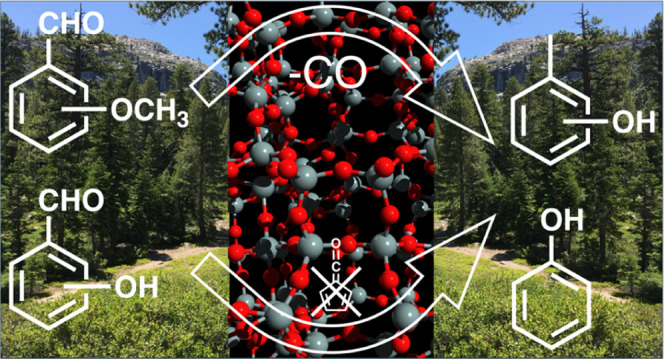

## Introduction

Tailored catalysts can selectively steer a chemical reaction toward a desired product. This is achieved by stabilizing or destabilizing transition states between reactants, intermediates, and products, which are responsible for driving selectivities and conversion in catalysis.^1^ On the one hand, open-shell radical species were found to play an important role in oxidative and nonoxidative coupling of methane,^2,3^ oxyhalogenation,^4,5^ and propane oxidative dehydrogenation.^6^ Closed-shell ketenes,^7,8^ on the other hand, enable the formation of the first olefins in methanol to olefin conversion and play a crucial role in biomass conversion over zeolites.^9–12^ Numerous elementary reactions of the lignin (catalytic) pyrolysis mechanism were elucidated in a bottom-up approach on the basis of model compounds, e.g., methoxyphenols,^13–15^ benzenediols,^16–18^ hydroxybenzaldehydes,^17^ and anisaldehydes,^19^ to approach the full complexity of the lignin chemistry in a stepwise manner. Condensation reactions favoring coke formation were proposed to be the result of quinone methide formation by Kawamoto et al.^20,21^ Fulvenone (*c*-C_5_H_4_=C=O), a reactive ketene, was found to play a major role in Wolff ring-contraction^22^ reactions as well as in lignin chemistry, depending on the substrate functionalization and the catalyst. This dependence provides opportunities to control the selectivity of this catalytic pyrolysis process.

Upon gas-phase pyrolysis, catechol (*ortho*-benzenediol, **R1**) and *ortho*-hydroxybenzaldehyde (**R2**) yield fulvenone (Scheme 1).^17,19^ The functional group interaction in the *ortho-*position [>C­(=O)–H···H–O<] is the driving force for efficient dehydration and dehydrogenation to ketene formation, which we refer to as *ortho* effect. Interestingly, this trend is reversed in anisaldehydes: After demethylation to aldehyde substituted phenoxy radicals, only the *meta* and *para* isomers decarbonylate (**R3**) and dehydrogenate to yield fulvenone. In *ortho*-anisaldehyde (**R4**), fulvenone is absent in gas-phase pyrolysis, which is rationalized by the calculated potential energy surface, where, after demethylation at the OCH_3_ group, a hydrogen shift from the aldehyde group to the phenoxy radical center initiates the decarbonylation. As a result, the 1-hydroxyphenyl radical rapidly (**R4**) rearranges to the phenoxy radical, the most stable C_6_H_5_O isomer.^19,23^ This makes aldehyde functionalities very potent reaction partners, with exceptional selectivity to fulvenone ketene during pyrolysis depending on the isomer used as reactant. Contrarily, neither methoxyphenols nor dimethoxybenzenes produce ketenes in gas-phase pyrolysis. Rapid demethylation (**R5**) occurs to the corresponding phenoxy radicals, which promptly decarbonylate yielding cyclopentadienone (*c*-C_5_H_4_=O, **R6**).^13,14,24^

**Scheme 1:**
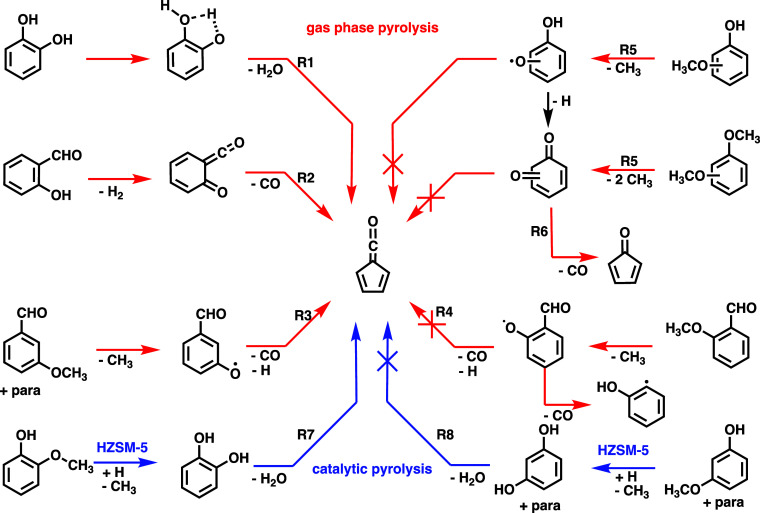
Comparison of Pathways Leading to Fulvenone (Center), Showing Active (Left) and Suppressed (Right) Channels, Utilizing Operando PEPICO Spectroscopy^a^

a Reactions are color coded: red = gas-phase pyrolysis, blue = catalytic pyrolysis over HZSM-5.

Upon adding a zeolite catalyst, specifically HZSM-5, fulvenone is formed in the catalytic pyrolysis of guaiacol and catechol (both are *ortho* substituted benzenes, **R7**), but not in the *meta* and *para* isomers of benzenediols or methoxyphenols (**R8**). The *meta* and *para* isomers are therefore less reactive and exhibit lower conversion.^19,25^ Although fulvenone is amenable to rapid hydrogenation to phenol in catalytic pyrolysis, it can also decarbonylate to cyclopentadiene, which is subsequently methylated to methylcyclopentadienes, the precursors of fulvene (*c*-C_5_H_4_=CH_2_). Furthermore, naphthalene and indene are generated via cyclopentadiene dimerization, limiting the catalyst lifetime due to deactivation via coking reactions. Utilizing faujasites (FAU) and increasing the Brønsted acid site density, the dehydration reaction to fulvenone can be suppressed, too, and, as a result, the phenol selectivity increases by a factor of 5.^1^ The high concentration of adjacent protons in the zeolite enables simultaneous surface coordination of both catechol OH groups, ultimately suppressing the *ortho* effect (dehydration to fulvenone by **R1**, **R7**) and enhancing dehydration to phenol.

Fulvenone ketene and its derivatives were also observed in anisole flames^26^ and in the gas-phase pyrolysis reaction network of vanillin,^27^ making them ubiquitous reactive intermediates in biomass conversion.^28,29^

For a targeted optimization of lignin catalytic pyrolysis to increase its economic viability, ketenes must be traced, and their provenance fully understood to suppress their formation. Thanks to *operando* photoelectron photoion coincidence (PEPICO) spectroscopy with vacuum ultraviolet (VUV) synchrotron radiation, fulvenone detection is now routinely possible and even quantification is within reach thanks to recent photoionization cross section measurements.^28,30^ PEPICO is a soft ionization tool, which suppresses dissociative ionization (fragmentation), leading to a single peak in the mass spectrum for each neutral component of the sample. This technique is universal as ionization is not limited by selections rules and practically all species desorbing from the surface can be detected if their ionization energy is below the photon energy used. The detection of photoelectrons as a second analytical dimension delivers an isomer-specific fingerprint to assign all species by utilizing either reference spectra or Franck–Condon spectral modeling as taken from the PEPISCO database.^31^ With this experiment, we have a tool to follow the formation of fulvenone ketene at *operando* conditions, verifying its absence or presence upon catalytic and gas-phase pyrolysis and its influence on the downstream mechanism.

Among the representative lignin monomers, aldehydes have received little attention in catalytic pyrolysis over zeolites, although they are potent reactants to yield fulvenone, and they may also undergo facile decarbonylation, which may be leveraged to deoxygenate lignin and to optimize the valorization process. Thus, the objective of this paper is to track the formation of reactive ketene species during catalytic pyrolysis of the lignin model compounds hydroxybenzaldehyde and anisaldehyde and ultimately understand their reaction mechanism over HZSM-5. Triggered by the gas-phase pyrolysis mechanism of *ortho*-hydroxybenzaldehyde (**R2**), we hypothesize that the *ortho* effect, where vicinal functional groups, in this case, aldehyde and hydroxy, interact preferably, initiates a dehydrogenation and decarbonylation to ketene also over zeolite catalysts, thanks to the abundant Brønsted acid sites. Thus, our goal is to reveal if fulvenone is formed influencing the selectivity of the process, or if other reaction channels are at play, which may be leveraged to optimize lignin catalytic pyrolysis.

## Results and Discussion

### Keeping an Eye on Ketene Formation

Mass spectra during the catalytic pyrolysis of *para*- and *ortho*-anisaldehyde over HZSM-5 are presented as a function of the reactor temperature in Figure 1. The spectra reveal similar products from the *para* and *ortho* isomers. No reaction is expected at 298 K, but a minor *m*/*z* 122 peak was still observed in the *para* isomer, indicating a sample impurity (Figure 1a). However, the intensity of the *m*/*z* 122 peak increased with temperature, suggesting it was also produced via catalytic pyrolysis. Although *ortho*-anisaldehyde exhibits minor peaks at *m*/*z* 118 and 119 (Figure 1b), they only arise from the dissociative photoionization of the *ortho*-anisaldehyde reactant.^31^ Additionally, mass peaks at *m*/*z* 108, 94, and 32 were detected at similar reaction temperatures for both reactant isomers. When the temperature was increased to 760 K, minor peaks at *m*/*z* 106, 92, and 78 emerged, as well. While the *m*/*z* 32 peak is easily assigned to methanol, the isomer-specific assignment of higher molecular weight products requires more information.

**Figure 1:**
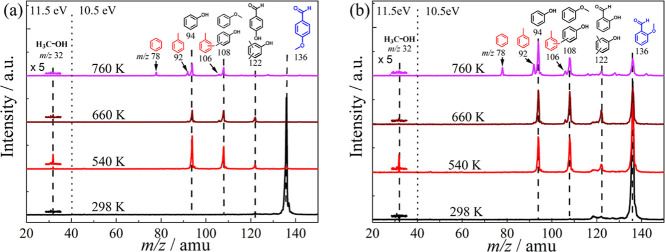
Time-of-flight mass spectra of (a) *para*-anisaldehyde and (b) *ortho*-anisaldehyde upon catalytic pyrolysis over HZSM-5 at different reactor temperatures. Mass spectra were recorded at *h* *ν* = 10.5 eV for the *m*/*z* > 40 range, and *h*ν = 11.5 eV, sufficiently high to ionize methanol (*m*/*z* 32), for *m*/*z* < 40.

Based on the mass-selected threshold photoelectron spectra in Figure 2, major peaks are identified as benzene (*m*/*z* 78), toluene (*m*/*z* 92), phenol (*m*/*z* 94), and anisole or methylphenols (cresols, *m*/*z* 108) for both *para*- (Figure 2a–e) and *ortho*-anisaldehyde (Figure 2f–j). Fulvenone is not identified in the reaction mixture, as the *m*/*z* 92 peak at the ionization energy of fulvenone and its characteristic vibrational resonances are missing. Although the *m*/*z* 122 ms-TPES is weak, it strongly suggests the presence of hydroxybenzaldehyde in *ortho*-anisaldehyde (Figure 2j) and likely dimethylphenol and methylanisole isomers because of the ionization onset at ca. 8 eV in *para*-anisaldehyde (Figure 2e). This is based on the abundance of cross-methylation products methylphenol and anisole (*m*/*z* 108), and the low, ca. 8 eV ionization energy of dimethylphenol isomers.^32^ In the absence of gaseous methyl radicals, ample surface CH_3_ species may participate in surface-confined cross-methylation pathways leading to dimethylphenol and methylanisole (*m*/*z* 122). On the other hand, the low hydroxybenzaldehyde (*m*/*z* 122) signal in Figures 1 and 2e,j suggests that its formation is inhibited because the decarbonylation of anisaldehyde competes effectively with demethylation over HZSM-5 or that all its isomers are highly reactive and promptly consumed prior to desorption from the surface. This leads to three questions: (1) Are decarbonylation and demethylation reactions competitive? (2) Are they surface-confined or radical-driven in the gas phase? (3) What is the mode of fulvenone suppression?

**Figure 2:**
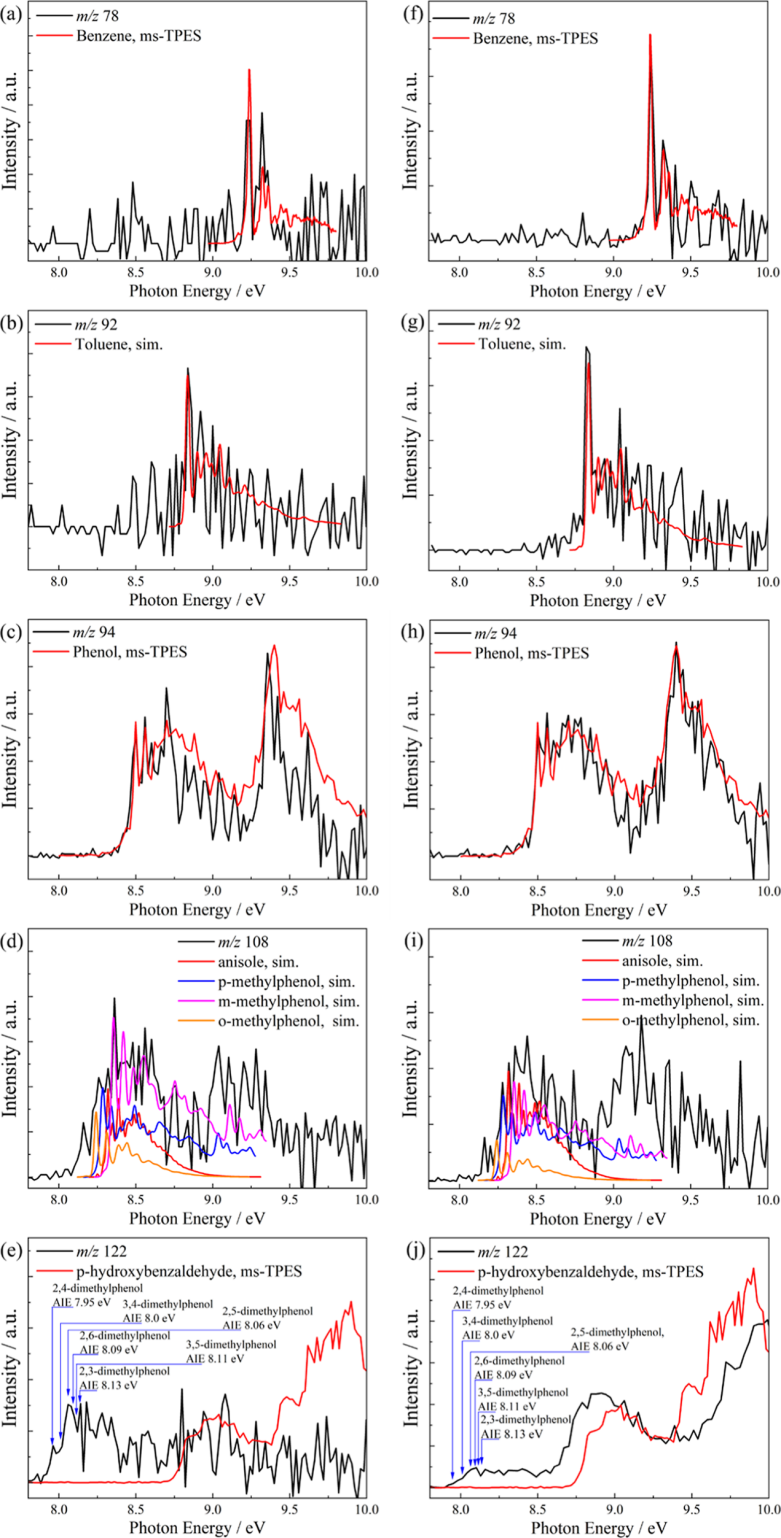
Mass-selected threshold photoelectron spectra of anisaldehyde catalytic pyrolysis products, compared with experimental reference spectra and Franck–Condon simulations for (a–e) *para*-anisaldehyde and (f–j) *ortho*-anisaldehyde.

### Radical- vs Acid-Catalyzed Reactions

To address the competition between the reactivity of the aldehyde and methoxy functional groups and whether the mechanism of CO or CH_3_ loss is radical-driven, we investigated the catalytic pyrolysis of benzaldehyde and anisole over HZSM-5 (Figure 3a,b). Benzaldehyde decarbonylation occurs at temperatures as low as 540 K, much below the 1300 K threshold for the radical-driven reaction with an activation energy of 364 kJ/mol.^33^ The intermediately formed phenylcarbonyl radical rapidly loses CO to yield phenyl radicals as found by Vasiliou et al. (Scheme 2, **R9**)^33^ We thus conclude that an acid-catalyzed reaction proceeds via carbocation formation at the phenyl ring (Scheme 2, **R10**). To rationalize this pathway, we calculated the potential energy surface of the decarbonylation of protonated benzaldehyde (Supporting Information, Figure S1), to mimic the reaction on the acid sites of HZSM-5. The decarbonylation of protonated benzaldehyde is preceded by hydrogen shifts over 5-membered ring transition states with a rate-limiting barrier of ca. 170 kJ/mol. Afterward, decarbonylation proceeds downhill to produce C_6_H_7_^+^, which yields benzene and recovers the proton of the zeolite. This mechanism is in good agreement with the experimental and theoretical work of Dopfer et al., who investigated protonated benzaldehyde. They found an ion–neutral complex of a formyl cation with benzene (see Figure S1 B7), effectively protonating benzene upon CO loss to afford C_6_H_7_^+^, in analogy to what happens on the Brønsted acid site.^34^ Although this protonation mechanism neglects the zeolite cage, the activation energy is almost 200 kJ/mol lower than that of the gas-phase pyrolysis reaction, rationalizing the much lower reaction temperature upon utilizing HZSM-5 in our experiments.

**Figure 3:**
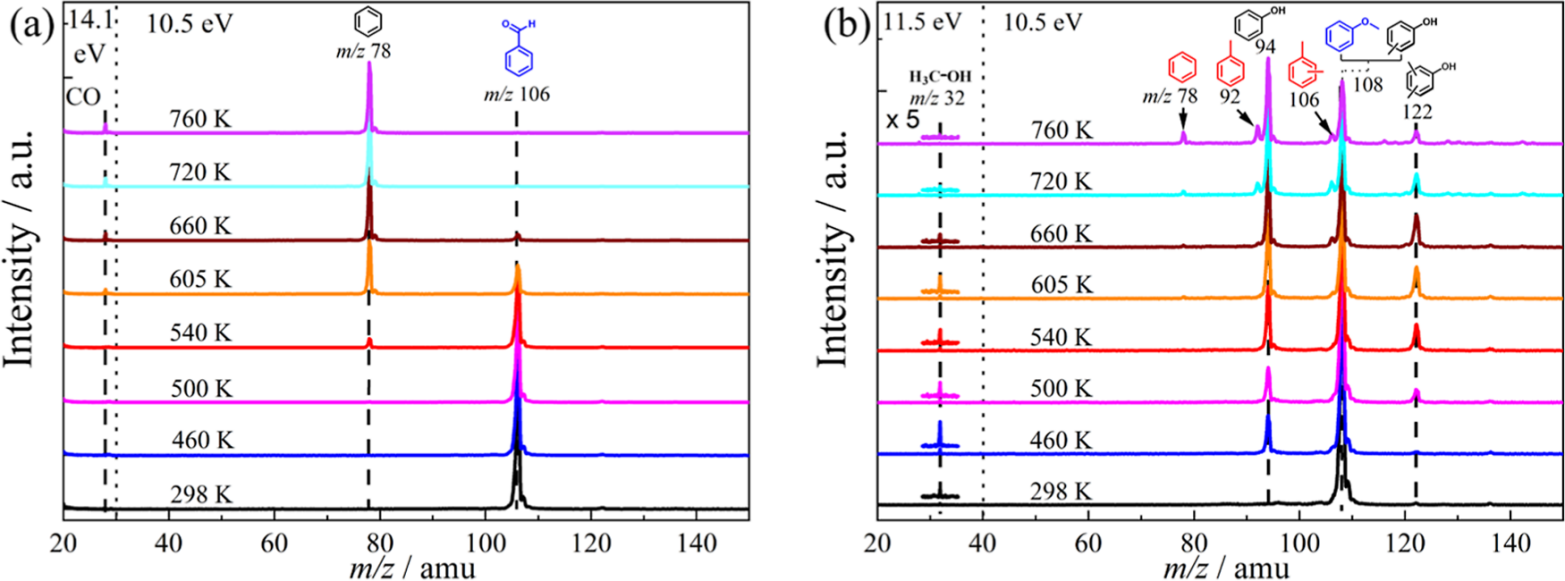
Time-of-flight mass spectra of (a) benzaldehyde and (b) anisole catalytic pyrolysis over HZSM-5 as a function of the reactor temperature. Mass spectra were recorded at hν = 14.1 eV below *m*/*z* 30 and 10.5 eV above *m*/*z* 30 for benzaldehyde and at hν = 11.5 eV below *m*/*z* 40 and 10.5 eV above *m*/*z* 40 to detect the high ionization energy species CO and methanol.

**Scheme 2:**
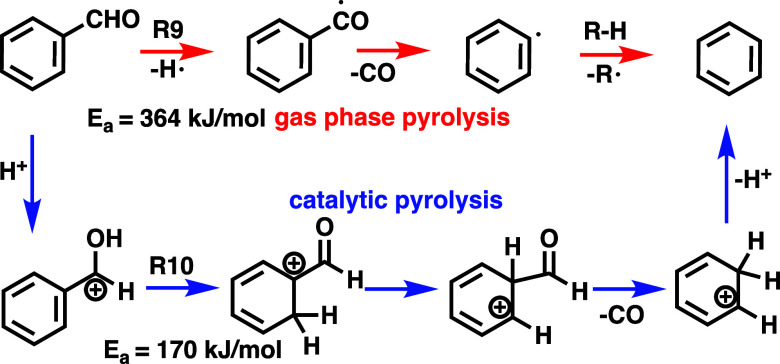
Mechanism of the Gas-phase Pyrolysis (Red Arrows)^33^ and the Acid-Catalyzed Pyrolysis of Benzaldehyde (Blue Arrows, Mimicking Catalytic Pyrolysis Over HZSM-5),^35^ of which the Latter Proceeds at Temperatures as Low as 540 K^a^

a Activation energies (*E*_a_) are taken from the literature^33^ or calculated herein using B3LYP/6−311++G­(d,p) (Supporting Information, Figure S1).

The decomposition (light-off) temperature of anisole was lower, at 460 K, as shown by the appearance of the *m*/*z* 94 and 122 peaks, than the ca. 540 K required for benzaldehyde conversion. Full conversion was, however, achieved at 720 K for benzaldehyde, at which temperature anisole still showed unconverted *m*/*z* 108 signal. Thus, the reactivity of the two functional groups of anisaldehyde is not substantially different in catalytic pyrolysis over HZSM-5. Demethylation is likely also acid-catalyzed (Scheme 3, **R11**), taking into account the high, 1300 K temperature in the noncatalytic experiments of Friderichsen et al.^36^ This is further confirmed by the absence of gaseous methyl and cyclopentadienyl radicals and cyclopentadiene in our experiment. (**R12**) Furthermore, rapid acid-catalyzed transmethylation is responsible for the formation of cresols (**R11**). In addition, the *m*/*z* 92 ms-TPES (Figure 2b,g) reveals toluene formation, likely produced via cresol dehydroxylation (*m*/*z* 108 → *m*/*z* 92) or via benzene methylation.

**Scheme 3:**
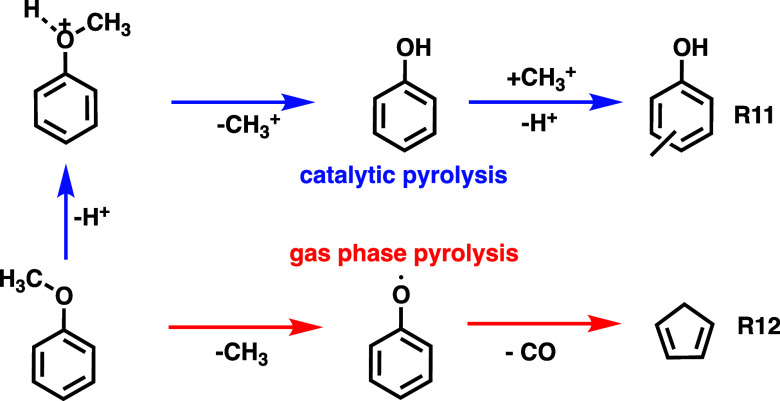
Mechanism of the Acid-Catalyzed Anisole Pyrolysis (Blue Arrows), which Proceeds at Temperatures as Low as 540 K, in Comparison with the Unimolecular Demethylation of Anisole^36^ from Friedrichsen et al. At Temperatures as High as 1300 K (Red Arrows)

Fulvenone ketene (*c*-C_5_H_4_=C=O), also at *m*/*z* 92, could not be identified in the ms-TPES, although it is formed in *para*-anisaldehyde (**R3**)^19^ and salicylaldehyde (**R2**) noncatalytic pyrolysis.^17^ This raises the question whether the demethylated species on the surface form hydroxybenzaldehydes (*m*/*z* 122), which yield fulvenone after dehydrogenation and decarbonylation in pyrolysis measurements (**R2**),^17^ or if ketene formation is outcompeted by alternative decarbonylation channels over HZSM-5. To address this question, we investigate the catalytic pyrolysis of hydroxybenzaldehyde isomers next.

### Dehydrogenation vs Decarbonylation in Hydroxybenzaldehyde

Catalytic pyrolysis mass spectra of *para*- and *ortho*-hydroxybenzaldehyde are depicted in Figure 4, which show mostly phenol (*m*/*z* 94) formation at 10.5 eV upon increasing the temperature. Full conversion of the reactant is observed at temperatures as low as 540 K. Therefore, the catalytic pyrolysis path of hydroxybenzaldehyde proceeds exclusively via decarbonylation of the aldehyde group to yield carbon monoxide and phenol. Intriguingly, fulvenone (*m*/*z* 92), the formation of which could be promoted by the *ortho* effect, is absent in the mass spectrum of Figure 4. It can thus be concluded that the intramolecular dehydrogenation to 2-carbonylcyclohexadienone, promoted by the interaction of adjacent functional groups (>C­(=O)–H···H–O<) to produce the precursor of fulvenone, is inhibited over HZSM-5, but the exact mechanisms remain elusive.

**Figure 4:**
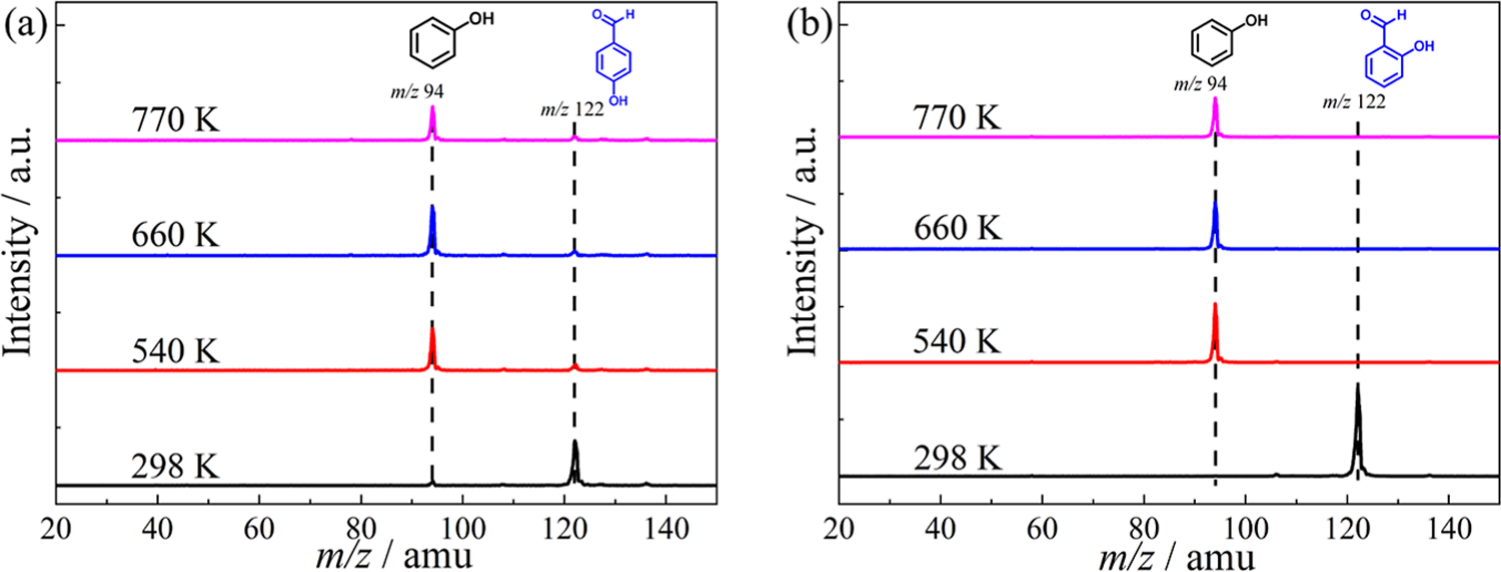
Time-of-flight mass spectra of (a) *para*-hydroxybenzaldehyde and (b) *ortho*-hydroxybenzaldehyde catalytic pyrolysis over HZSM-5 at representative temperatures recorded at *hν* = 10.5 eV.

Thus, building upon the acid catalyzed decarbonylation mechanisms of benzaldehyde (Scheme 2 and Figure S1), we calculated the decarbonylation of protonated salicylaldehyde and compared it to the dehydrogenation mechanism, which is driven by the *ortho* effect. While both pathways (Figure 5) follow the same initial hydrogen transfer reactions **A1** to **A5**, a branching occurs, which either proceeds downhill **A5** → **A6** ^‡^ → **A10** to yield CO and protonated phenol (**A10**, black pathway) or follows several torsions of the CHO or OH groups to initiate dehydrogenation at the CH_2_ group of the benzene ring and at the aldehyde group. This structure undergoes H_2_ loss to form **A16** over a barrier of 317 kJ/mol, which cannot compete with the direct decarbonylation to produce **A10**. This renders the reaction to protonated fulvenone (**A19**) unlikely and refutes our initial hypothesis that the *ortho* effect plays a role in the presence of HZSM-5.

**Figure 5:**
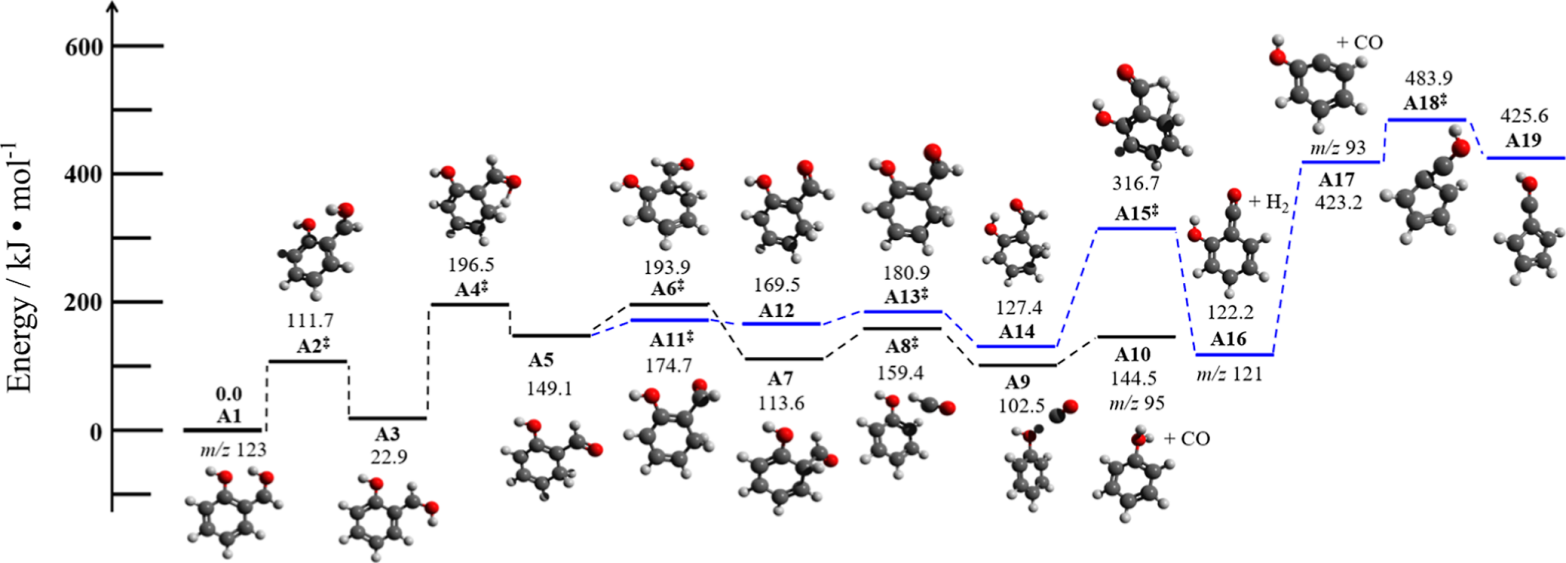
Reactions of protonated salicylaldehyde mimicking acid-catalyzed decarbonylation and dehydrogenation over HZSM-5 at B3LYP/6−311++G­(d,p) level of theory. Hydrogen transfer reactions initiate the chemistry, with an overall barrier of 197 kJ/mol to protonated phenol and CO. The dehydrogenation (blue pathway) requires 317 kJ/mol to yield the ketene **A16** and another 362 kJ/mol to yield protonated fulvenone **A19**, rendering this reaction unfeasible.

With the suppression of fulvenone now understood, we focus on alternative demethoxylation channels over HZSM-5, as small methanol signals were detected at *m*/*z* 32 during anisaldehyde and anisole catalytic pyrolysis (Figure 1), to provide a full picture of the catalytic pyrolysis mechanism of these model compounds.

### The Role of Demethoxylation

Both anisaldehyde (Figure 1) and anisole (Figure 3b) have methoxy groups and the coproduct of anisaldehyde demethoxylation would be benzaldehyde at *m*/*z* 106. However, the *m*/*z* 32 peak was already observed at 460 K, while the *m*/*z* 106 signal was only observed above 660 K (see Figure 1). Although the signal-to-noise ratio of *m*/*z* 106 peak is insufficient to identify the species via ms-TPES, the benzaldehyde catalytic pyrolysis results provide circumstantial evidence about its identity. Benzaldehyde reaches near full conversion at temperatures of 660 K (see Figure 3a). Consequently, the appearance of *m*/*z* 106 above 660 K suggests the presence of xylene rather than benzaldehyde. This indicates that methanol does not originate from the methoxy group of anisaldehyde. Additionally, anisole pyrolysis showed a similar temperature to produce methanol (*m*/*z* 32) as the anisaldehyde results, while the phenol coproduct at *m*/*z* 94 was observed simultaneously (see Figure 3b). These findings suggest that CH_3_OH originates from anisole rather than from the reactant, anisaldehyde. Thus, the demethoxylation channel is outcompeted by demethylation and decarbonylation in anisaldehyde isomers (Scheme 4, **R13**).

**Scheme 4:**
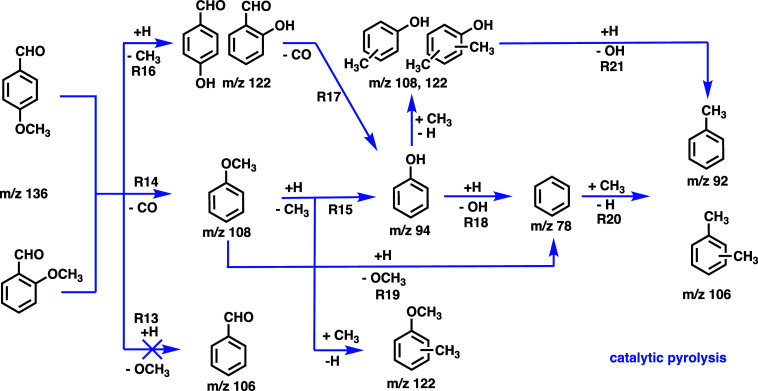
Anisaldehyde and Salicylaldehyde Catalytic Pyrolysis Pathways Over HZSM-5^a^

a For clarity, only reaction intermediates and products are shown, while surface intermediates (protonated species) are not presented.

### Reaction Mechanism of Anisaldehydes

Based on the analysis of intermediates and products and the resulting submechanisms, the main reaction pathways for the catalytic pyrolysis of anisaldehyde is summarized in Scheme 4. These reactions are acid catalyzed throughout, as shown in the submechanisms in Schemes 2 and 3, respectively. There are two initial decomposition reactions: one involves decarbonylation to produce anisole at *m*/*z* 108 (**R14**), followed by demethylation to phenol *m*/*z* 94 (**R15**). The other pathway starts with demethylation to intermediately generate hydroxybenzaldehyde *m*/*z* 122 (**R16**), which rapidly undergoes decarbonylation to yield phenol *m*/*z* 94 (**R17**), which is responsible for the fulvenone suppression (absence of the *ortho* effect). As the temperature increases, phenol and anisole will dehydroxylate (**R18**) or demethoxylate (**R19**), respectively, to produce benzene, which may subsequently undergo methylation to toluene *m*/*z* 92 and xylene *m*/*z* 106 (**R20**). It is noteworthy that toluene and xylene already appear at 660 K, whereas benzene is only observed at 760 K (see Figure 1). This suggests that benzene is formed via dehydroxylation of phenol (**R18**) requiring a higher activation energy, as compared to the dehydroxylation of cresols and dimethylphenols (**R21)** yielding toluene and xylene, respectively. We conclude that methyl substitution lowers the activation energy of the dehydroxylation (**R21**).

These mechanistic insights call for a comparison of methoxyphenols and benzenediols, both of which form fulvenone (Scheme 5) with the anisaldehydes to evaluate opportunities to optimize selectivities.

**Scheme 5:**
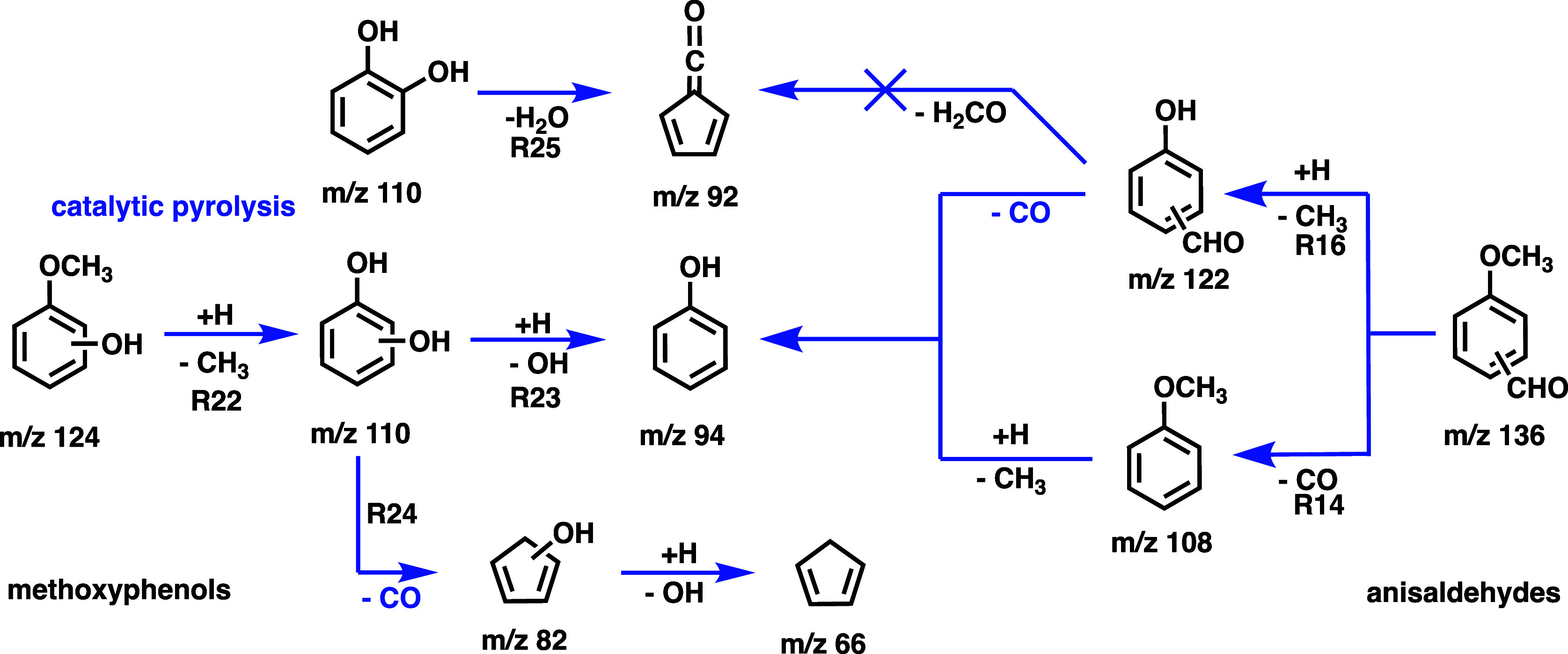
Comparison of the Catalytic Pyrolysis Pathways of Anisaldehydes and Methoxyphenols Over HZSM-5^a^

a For clarity, only reaction intermediates and products are shown, while surface intermediates (protonated species) are not presented.

### Assessing the Reactivity of the Hydroxyl and Aldehyde Groups

In previous studies,^29,37^ we found that methoxyphenol catalytic pyrolysis is initiated by demethylation, yielding surface-bound hydroxy-phenoxy species. Subsequently, benzenediols are formed upon hydrogen abstraction from the zeolite surface (**R22,** Scheme 5).^19^ Benzenediol isomers share two primary catalytic pyrolysis pathways: the first one involves dehydroxylation (**R23**) and hydrogen addition on the zeolite surface to form phenol. The second one proceeds via decarbonylation to produce hydroxycyclopentadiene (**R24**), followed by dehydroxylation and hydrogen addition to yield cyclopentadiene.^19,25^ *Ortho*-benzenediol (**R25**) is subject to the *ortho* effect and also exhibits a dehydration path facilitated by the interaction of adjacent hydroxyl groups, forming fulvenone ketene.^25^ This intermediate is responsible for the high reactivity and low selectivity over HZSM-5, but its formation can be suppressed by utilizing HFAU with a high Brønsted acid site density.

The catalytic pyrolysis of anisaldehyde is also initiated via demethylation (**R16**) or decarbonylation (**R14**), resulting in the production of hydroxybenzaldehyde and anisole, which further decarbonylate and demethylate, respectively, to produce phenol. No fulvenone was observed during the catalytic pyrolysis of anisaldehyde over HZSM-5, indicating that the adjacent hydroxyl and aldehyde groups do not interact on the surface to facilitate dehydrogenation, and the decarbonylation reaction proceeds instead. Rapid decarbonylation is responsible for the phenol and cresol selectivity increase in anisaldehydes, which is clearly visible by direct comparison to guaiacol (see Figure 6). This makes the chemistry of anis- and hydroxybenzaldehydes over HZSM-5 distinct from the unimolecular pyrolysis in the gas phase (**R2**, **R3**) and offers an opportunity to control lignin pyrolysis chemistry as outlined in the conclusion.

**Figure 6:**
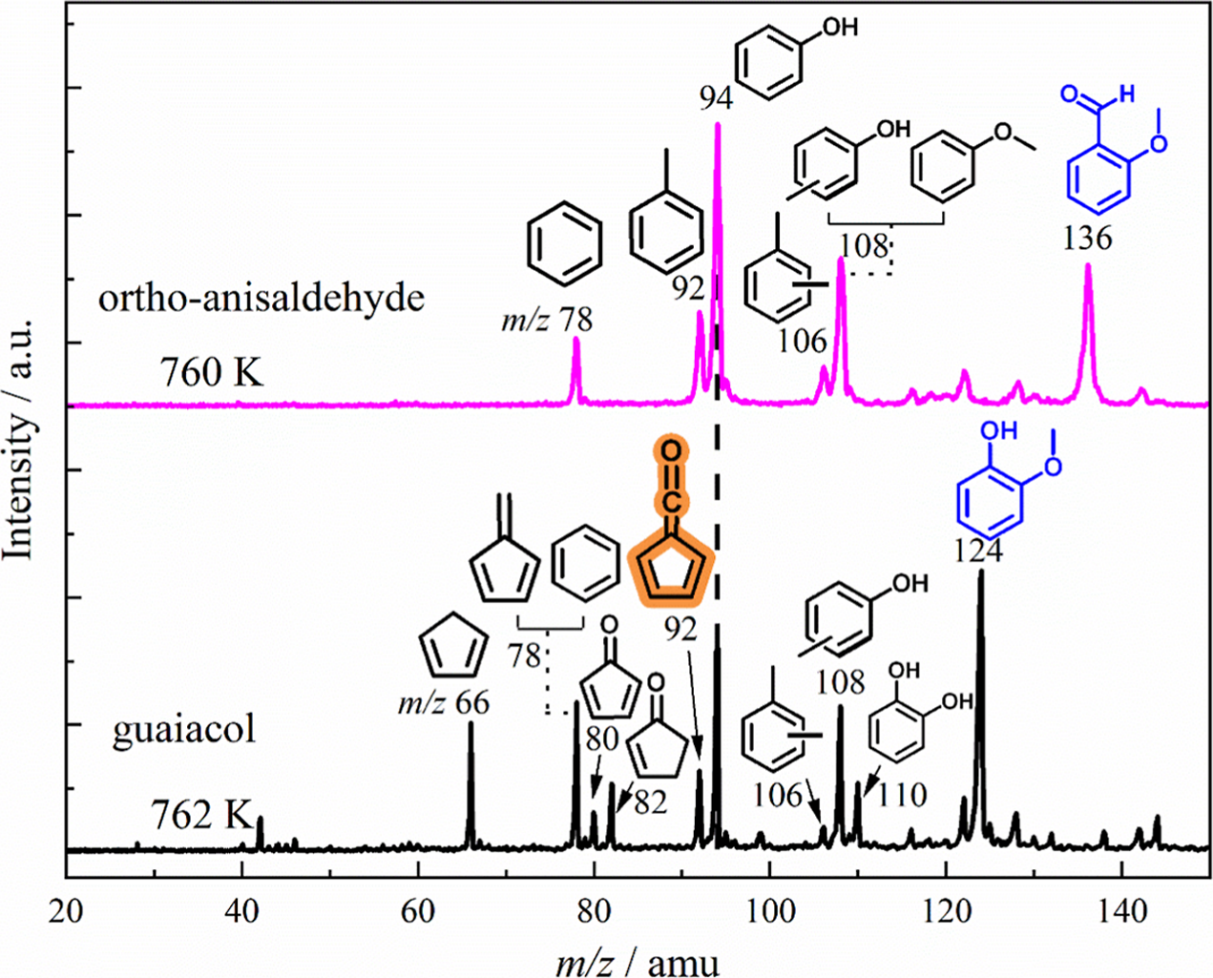
Comparison of guaiacol (*ortho*-methoxyphenol)^37^ and *ortho*-anisaldehyde catalytic pyrolysis mass spectra under similar conditions. Due to fast decarbonylation, the product selectivity to phenol and methylphenols (cresols) is higher in *ortho*-anisaldehyde (upper trace).

## Summary and Conclusions

In this work, we unveiled the catalytic pyrolysis mechanism of anisaldehydes and hydroxybenzaldehydes to understand their pyrolysis chemistry to form the fulvenone ketene, a reactive intermediate, often observed in lignin chemistry and responsible for limiting selectivity and catalyst lifetime. *Operando* PEPICO spectroscopy was employed to record mass spectra and photoion mass-selected TPES to isomer-selectively assign intermediates and products. The mechanism was solved by understanding the individual submechanisms: (1) The reactivity of methoxy and aldehyde groups, as studied by anisole and benzaldehyde model compounds, revealed that the reaction is surface confined, as no radicals could be observed in the gas phase. (2) By utilizing hydroxybenzaldehyde, we uncovered that surface-catalyzed decarbonylation to phenol outcompetes the *ortho* interaction of the > C­(=O)–H and HO–C< groups, suppressing dehydrogenation and ketene formation, readily observed without catalyst. DFT calculations indicated that the acid-catalyzed decarbonylation is much less endothermic than the dehydrogenation to yield fulvenone. (3) Furthermore, we evaluated potential demethoxylation reactions in anisaldehydes over HZSM-5 to complete the reaction mechanism.

In summary, the HZSM-5 catalyzed pyrolysis of both *para*- and *ortho*-anisaldehyde is initiated by either demethylation to yield the respective hydroxybenzaldehydes, followed by decarbonylation to form phenol, or by initial decarbonylation to form anisole, followed by demethylation to phenol over HZSM-5 zeolite. At higher temperatures, phenol undergoes dehydroxylation to benzene. Surface-bound methyl groups readily methylate products and intermediates revealing rich methyl-transfer chemistry. Fulvenone is absent in the catalytic pyrolysis of anisaldehydes. We attribute this to the facile decarbonylation of the aldehyde functionality, which is faster than elimination reactions promoted by the interaction of vicinal functional groups, observed in, e.g., *ortho*-hydroxybenzaldehyde during noncatalytic pyrolysis, where intramolecular dehydrogenation produces 2-carbonyl cyclohexadienone (**R2**), the precursor of fulvenone.

Fast decarbonylation in anis- and methoxybenzaldehydes can help lignin valorization strategies to increase the selectivity to phenols over zeolite catalysts. During the initial depolymerization of lignin, aldehyde groups can serve as target functional groups due to their low catalytic pyrolysis temperature (ca. 500 K) leading to direct decarbonylation, which may reduce byproduct and coke formation. Methoxy groups, on the other hand, are more prone to demethylation to generate phenols. Surface methyl groups participate extensively in cross-methylation, which provides opportunities to lower the deoxygenation temperature of methylated phenols (cresols, xylenols) in comparison to bare phenol. Moreover, methylation reactions can be suppressed by utilizing FAU catalysts, which also have the ability to reduce ketene formation, but are generally deactivated faster.^1,29^ Hydroxyl groups, in comparison to aldehyde or methoxy functionalities, react at higher temperatures over HZSM-5, which also provides opportunities for pathway control to generate target products by tuning the temperature and selecting the target functional groups.

## Experimental and Computational

The experiments were performed at the VUV beamline of the Swiss Light Source,^38^ Paul Scherrer Institute using the double imaging photoelectron photoion coincidence (CRF-PEPICO) spectrometer.^39^ Synchrotron radiation was delivered from a bending magnet port and the light was collimated, dispersed by a grazing incidence monochromator with a 150 lines·mm^–1^ blazed grating, and focused at a 200 μm exit slit in a differentially pumped rare gas filter. The latter was filled with a mixture of krypton, argon and neon or a mixture of argon and neon, at a pressure of 10 mbar over an optical length of 10 cm to suppress higher-order radiation above 13.9 and 15.5 eV, respectively. The photon beam intersected the sample ca. 50 cm downstream from the focus with an energy resolution of 2–8 meV, depending on the photon energy. The photon energy was calibrated using the Ar 11s′–13s′ autoionization lines in the first and second order of the grating.

Ten mg of HZSM-5 (Zeolist, average pore diameter 5.4–5.6 Å, *V*_micro_ = 0.15 cm^3^/g, *S*_BET_ 364 m^2^/g, BAS = 0.33 mmol/g, LAS = 0.064 mmol/g),^1^ sandwiched between quartz wool, was packed in a temperature-controlled quartz tube reactor with a 2 mm inner diameter, 4 mm outer diameter, and 26 mm heated length. The sample was placed in a stainless steel container, which was temperature controlled by a thermostat to set the vapor pressure of the reactant and the resulting dilution to typically 0.1% at a total argon flow of 20 sccm. The gas flow passed through the heated reactor and exited it through a 2 mm diameter nozzle into the high vacuum of the source chamber. The remaining unconverted sample, intermediates and products desorbed from the catalyst thus formed a molecular beam, which was sampled by a 2 mm skimmer. After traveling through the skimmer, the molecular beam was photoionized by monochromatic synchrotron radiation. Photoions and photoelectrons were extracted in opposite directions and detected at the end of two flight tubes utilizing fast imaging delay line detectors (Roentdek DLD40). Time-of-flight photoionization mass spectra are obtained based on delayed coincidences between electrons and ions. Mass discrimination factors as well as the ion collection efficiencies were found to be virtually constant across the investigated *m*/*z* range.^40^ In addition, a sensitivity of <1 ppm^41^ ensures detection of all species, if the photon energy exceeds the adiabatic ionization energy. Product and intermediate quantification to extract branching ratios is challenging, due to unknown photoionization cross sections. Signal levels and abundant dissociative ionization at the high photon energy required to ionize CO puts its measurements beyond the dynamic range of the experiment, but the coproducts of decarbonylation reactions are detected, which allowed us to use mass spectral peak intensities in a semiquantitative analysis.

Electron velocity map imaging allows for electron kinetic energy analysis and thereby plotting photoion mass-selected threshold photoelectron spectra (ms-TPES). On the basis of reference or simulated photoelectron spectra, the pyrolysis products were identified.^42^ For species without literature reference spectra, Franck–Condon factors were calculated for the ionizing transitions between the neutral and the ground electronic state of the cation at room temperature within the double harmonic approximation.

Quantum chemical calculations were conducted by the Gaussian 16 (rev. A.03 & C.01) software.^43^ Franck–Condon simulations of the photoelectron spectra were carried out using the optimized geometries and vibrational frequencies at the B3LYP/6−311++G­(d,p) level, and adiabatic ionization energies were computed using the G4 composite method.^44^ The potential energy surface was explored by performing constrained coordinate scans and synchronous transit-guided quasi-Newton calculations to find stationary points.

## Supplementary Material



## Data Availability

The data underlying this study are available in the published article and its Supporting Information.

## Notes

It contains DFT and composite method calculations supporting our work (PDF)
