# Supplementary material for: Facile Decarbonylation Suppresses the Ortho Effect and Ketene Formation in the Catalytic Pyrolysis of Substituted Benzaldehyde Lignin Model Compounds
Source: J Org Chem. 2025 Sep 25;90(39):13802–11. doi: 10.1021/acs.joc.5c01411 (PMC12501935; doi:10.1021/acs.joc.5c01411)
Supplement: Supplementary file 1 [file jo5c01411_si_001.pdf]

Supplementary Information

Facile decarbonylation suppresses the *ortho* effect and ketene formation in the catalytic pyrolysis of substituted benzaldehyde lignin model compounds

Xiangkun Wu,[a,b] Zeyou Pan,[b] Zihao Zhang,[b] Keyong Hou,[a] Saša Bjelić,[b] Andras Bodi,[b] and Patrick Hemberger[b,*]

[a] *Environment Research Institute, Shandong University, 266237 Qingdao, China*

[b] *Paul Scherrer Institute, 5232 Villigen, Switzerland*

[*] Author to whom correspondence should be addressed:

Electronic mail: patrick.hemberger@psi.ch

Contents:

Fig. S1. Protonated benzaldehyde decarbonylation pathway at B3LYP/6-311++G(d,p) level theory.

Table S1. Ionization energy (IE) Anisaldehyde catalytic fast pyrolysis products.

Table S2. Optimized structures at B3LYP/6-311++G(d,p) level.

Table S3. Optimized structures by G4 method.

References

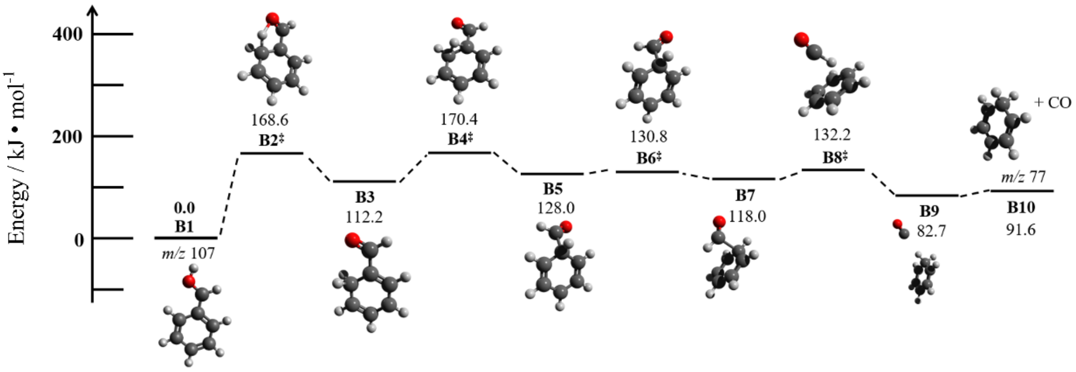

Fig. S1 Protonated benzaldehyde decarbonylation pathway at B3LYP/6-311++G(d,p) level theory, which is in agreement with literature findings.[1-7]

Table S1. Ionization energies (IE) of anisaldehyde catalytic fast pyrolysis products.

compound	*m/z*	Calculated IE (eV)	
		B3LYP/6-31G(d,p)	G4
Toluene	92	8.59	8.85
Anisole	108	8.03	8.25
p-Methylphenol	108	7.95	8.16
m-Methylphenol	108	8.12	8.33
o-Methylphenol	108	8.07	8.28
Phenol, 2,3-dimethyl	122	7.92	8.13
Phenol, 2,4-dimethyl	122	7.75	7.95
Phenol, 2,5-dimethyl	122	7.87	8.06
Phenol, 2,6-dimethyl	122	7.88	8.09
Phenol, 3,4-dimethyl	122	7.79	7.99
Phenol, 3,5-dimethyl	122	7.92	8.11

Table S2. Optimized structures at B3LYP/6-311++G(d,p) level.

Toluene

Atomic	Coordinates (Angstroms)		
Number	X	Y	Z
6	-1.902699	-0.000043	0.008416
6	-1.199537	-1.203496	0.002085
6	0.194213	-1.200557	-0.008918
6	0.913117	0.000063	-0.01125
6	0.194138	1.2006	-0.008916
6	-1.199644	1.203438	0.002084
1	-2.986839	-0.00009	0.01398
1	-1.735912	-2.146176	0.001615
1	0.731807	-2.143672	-0.017973
1	0.731662	2.143749	-0.017971
1	-1.73608	2.146083	0.001611
6	2.423116	0.000026	0.009278
1	2.801538	-0.003643	1.037513
1	2.828822	-0.882574	-0.490848
1	2.828779	0.886132	-0.484603

Toluene Cation

Atomic	Coordinates (Angstroms)		
Number	X	Y	Z
6	-1.881111	0.000007	0.006549

6	-1.181625	-1.238595	0.001411
6	0.186442	-1.240269	-0.007602
6	0.91581	-0.000011	-0.015752
6	0.186449	1.240259	-0.007603
6	-1.181618	1.238601	0.001411
1	-2.96575	0.00001	0.016406
1	-1.739351	-2.167029	0.00379
1	0.740861	-2.171447	-0.012273
1	0.740878	2.171432	-0.012273
1	-1.739334	2.167041	0.00379
6	2.390922	-0.000004	-0.002337
1	2.723941	0.000209	1.052733
1	2.813582	-0.899333	-0.454166
1	2.813557	0.899186	-0.454478

Anisole

Atomic	Coordinates (Angstroms)		
Number	X	Y	Z
6	-0.452186	-0.271655	-0.000007
6	0.49662	-1.302077	-0.000001
6	-0.031412	1.060877	-0.000011
6	1.850832	-0.998437	0.000002
6	2.281939	0.331321	-0.000001
6	1.336031	1.34998	-0.000007

1	0.147373	-2.327636	0.000001
1	2.576438	-1.804295	0.000007
1	1.654334	2.386699	-0.00001
1	3.339921	0.564927	0.000001
1	-0.746617	1.872344	-0.000018
8	-1.759005	-0.669618	-0.000014
6	-2.773126	0.324835	0.000028
1	-2.716334	0.955262	0.89424
1	-3.718882	-0.214699	0.000051
1	-2.71639	0.955282	-0.894172

Anisole Cation

Atomic	Coordinates (Angstroms)		
Number	X	Y	Z
6	-0.450317	-0.254007	0.000002
6	0.513151	-1.323009	-0.000007
6	-0.029108	1.112183	0.000001
6	1.851028	-1.024794	-0.00001
6	2.264559	0.324123	-0.000008
6	1.314825	1.381271	-0.000004
1	0.140083	-2.339956	-0.000007
1	2.589195	-1.816999	-0.000014
1	1.662555	2.407191	-0.000005
1	3.322021	0.562439	-0.000012

1	-0.755318	1.913611	0.000003
8	-1.695475	-0.645124	0.000005
6	-2.81842	0.283403	0.000016
1	-2.784947	0.895451	0.901801
1	-3.699079	-0.351155	0.000071
1	-2.785013	0.895389	-0.901813

p-Methyphenol

Atomic	Coordinates (Angstroms)		
Number	X	Y	Z
6	-1.438909	-0.017431	0.001292
6	-0.749591	1.193064	-0.000669
6	-0.726181	-1.217597	-0.000457
6	0.645935	1.197937	-0.003503
6	0.662842	-1.193884	-0.003132
6	1.379593	0.010763	-0.003678
1	1.167229	2.149735	-0.006242
1	-1.296052	2.132011	-0.00162
8	-2.808224	-0.094751	0.002998
1	-3.18258	0.792043	0.002827
1	1.203065	-2.135606	-0.005635
1	-1.272175	-2.153419	-0.001046
6	2.889805	0.01534	0.004312
1	3.285839	-0.391549	0.940667

1	3.283415	1.027811	-0.107912
1	3.296088	-0.592172	-0.810018

p-Methyphenol Cation

Atomic	Coordinates (Angstroms)		
Number	X	Y	Z
6	1.432971	0.009506	0.002218
6	0.735766	-1.235363	-0.001417
6	0.720449	1.249039	-0.001639
6	-0.630375	-1.227659	-0.01023
6	-0.645831	1.232471	-0.009509
6	-1.366388	0.001375	-0.013956
1	-1.175407	-2.164423	-0.01542
1	1.292346	-2.166672	-0.000095
8	2.744182	0.095678	0.008228
1	3.189324	-0.767288	0.009402
1	-1.197703	2.164839	-0.01482
1	1.290712	2.169965	-0.000149
6	-2.850576	-0.015363	0.005929
1	-3.19421	-0.138779	1.04555
1	-3.254899	-0.862571	-0.552934
1	-3.27971	0.915465	-0.365733

m-Methyphenol

Atomic	Coordinates (Angstroms)		
Number	X	Y	Z
6	-1.213927	-0.367713	0.001153
6	-1.316689	1.02336	0.00777
6	0.038782	-0.981655	-0.010515
6	-0.152615	1.789586	0.0023
6	1.205484	-0.216407	-0.012088
6	1.09862	1.180723	-0.0086
1	0.083995	-2.064979	-0.019438
1	1.996158	1.789369	-0.016628
1	-0.227993	2.871345	0.002847
1	-2.291854	1.501265	0.012879
6	2.558366	-0.886654	0.009852
1	2.521294	-1.877257	-0.44861
1	2.914009	-1.013624	1.038229
1	3.304785	-0.292507	-0.522515
8	-2.311843	-1.188178	0.001288
1	-3.113775	-0.655631	0.003691

m-Methyphenol Cation

Atomic	Coordinates (Angstroms)		
Number	X	Y	Z
6	1.20301	-0.407796	-0.000035
6	1.362201	1.02033	0.000015

6	-0.081683	-1.016205	-0.000065
6	0.237915	1.801529	0.000053
6	-1.209326	-0.232511	-0.000055
6	-1.037913	1.193823	0.000072
1	-0.131064	-2.098243	-0.000116
1	-1.920766	1.82441	0.000223
1	0.318554	2.881331	0.000075
1	2.356858	1.453363	0.000024
6	-2.591512	-0.807185	-0.000036
1	-2.577427	-1.896234	-0.001769
1	-3.147954	-0.46422	-0.87842
1	-3.146649	-0.467079	0.880307
8	2.232772	-1.22244	-0.000005
1	3.090122	-0.765715	0.000019

o-Methyphenol

Atomic	Coordinates (Angstroms)		
Number	X	Y	Z
6	-0.410997	0.751839	0.000013
6	0.863476	1.316182	-0.000006
6	-0.589048	-0.640717	-0.000005
6	1.989846	0.494997	-0.000021
6	0.555957	-1.438041	-0.000039
6	1.838618	-0.887911	-0.000038

1	2.707846	-1.534821	-0.000054
1	2.977951	0.940764	-0.000028
1	0.974375	2.397084	-0.000021
8	-1.548914	1.52034	0.000011
1	-1.309206	2.452644	0.000415
1	0.436164	-2.516716	-0.000057
6	-1.976455	-1.226523	0.000022
1	-2.544063	-0.901593	-0.876967
1	-2.543935	-0.901784	0.877167
1	-1.936201	-2.31726	-0.000097

o-Methyphenol Cation

Atomic	Coordinates (Angstroms)		
Number	X	Y	Z
6	-0.357082	0.791339	0.000035
6	0.971009	1.297291	0.000024
6	-0.64381	-0.635965	-0.000012
6	2.010234	0.41052	-0.000032
6	0.446379	-1.492441	-0.00007
6	1.750661	-0.994983	-0.000079
1	2.587638	-1.683647	-0.000123
1	3.033806	0.764439	-0.000043
1	1.139673	2.368817	0.000059
8	-1.410898	1.576768	0.00009

1	-1.186275	2.522041	0.000121
1	0.280611	-2.562709	-0.000108
6	-2.056539	-1.106013	0.000014
1	-2.593409	-0.72393	-0.875161
1	-2.593275	-0.724183	0.875386
1	-2.106695	-2.193457	-0.000131

Phenol, 2,3-dimethyl

Atomic	Coordinates (Angstroms)		
Number	X	Y	Z
6	-1.225905	-0.028441	-0.000045
6	-1.329783	1.361253	-0.000029
6	0.020702	-0.675326	-0.000044
6	-0.171773	2.125843	0.00001
6	1.187975	0.113107	-0.000013
6	1.076181	1.506763	0.000012
8	-2.40592	-0.729207	0.00008
6	0.057602	-2.187032	0.000033
1	-0.438757	-2.606031	-0.884673
1	-0.436558	-2.605889	0.886025
1	1.075516	-2.57142	-0.001277
1	-2.229773	-1.674904	-0.000671
1	-2.314079	1.813267	-0.000035
1	-0.240991	3.207826	0.000032

1	1.976834	2.110515	0.000044
6	2.563179	-0.516711	0.000037
1	3.335587	0.253977	0.000158
1	2.725324	-1.145137	-0.88149
1	2.725184	-1.145292	0.881477

Phenol, 2,3-dimethyl Cation

Atomic	Coordinates (Angstroms)		
Number	X	Y	Z
6	-1.212823	-0.162577	-0.000036
6	-1.490341	1.244493	-0.000016
6	0.121956	-0.701002	-0.000058
6	-0.437984	2.111979	0.000011
6	1.175273	0.201749	0.000015
6	0.881168	1.597857	0.000029
8	-2.275625	-0.934872	-0.000008
6	0.294094	-2.194074	-0.000047
1	-0.159913	-2.651258	-0.887793
1	-0.158131	-2.650993	0.888748
1	1.342788	-2.479468	-0.001088
1	-2.060815	-1.882213	-0.000051
1	-2.525169	1.563223	-0.000018
1	-0.59852	3.18262	0.000025
1	1.711279	2.295645	0.000058

6	2.614866	-0.237355	0.000084
1	3.287234	0.619731	0.000272
1	2.84458	-0.842229	-0.881667
1	2.844417	-0.842504	0.881685

Phenol, 2,4-dimethyl

Atomic	Coordinates (Angstroms)		
Number	X	Y	Z
6	-1.112364	-0.637746	0.003854
6	-0.127395	-1.623354	-0.002067
6	-0.762431	0.719689	-0.000182
6	1.215112	-1.264903	-0.009446
6	0.597603	1.045441	-0.007624
6	1.605062	0.078737	-0.009625
1	0.874241	2.09601	-0.013776
8	-2.418866	-1.061468	0.008398
6	-1.82679	1.790145	-0.001952
1	-2.469583	1.733154	0.885323
1	-2.474243	1.725719	-0.885324
1	-1.376691	2.78382	-0.007171
1	-3.010896	-0.303185	0.01075
1	-0.431202	-2.66348	-0.004266
1	1.970457	-2.044306	-0.016929
6	3.064361	0.467977	0.011224

1	3.206455	1.498512	-0.322229
1	3.659271	-0.179202	-0.63888
1	3.484152	0.388777	1.020223

Phenol, 2,4-dimethyl Cation

Atomic	Coordinates (Angstroms)		
Number	X	Y	Z
6	1.0905	-0.66061	0.000003
6	0.070843	-1.656874	-0.000003
6	0.778743	0.749169	-0.000032
6	-1.23565	-1.269859	-0.000021
6	-0.554293	1.091219	-0.000054
6	-1.587556	0.119464	-0.000038
1	-0.830025	2.139447	-0.000097
8	2.328736	-1.105719	0.000052
6	1.8836	1.763496	0.000005
1	2.520521	1.666148	-0.887323
1	2.519723	1.66687	0.887988
1	1.481515	2.775586	-0.0006
1	2.990994	-0.396172	0.000029
1	0.371292	-2.697347	0.000007
1	-2.024153	-2.013378	-0.000028
6	-3.019908	0.517184	0.000021
1	-3.156379	1.597287	-0.000631

1	-3.530277	0.09469	0.874959
1	-3.530779	0.093488	-0.874021

Phenol, 2,5-dimethyl

Atomic	Coordinates (Angstroms)		
Number	X	Y	Z
6	-0.672117	0.790809	-0.000677
6	0.697898	1.041719	-0.009239
6	-1.171963	-0.521033	0.003584
6	1.618781	-0.007046	-0.010788
6	-0.239682	-1.560219	-0.001972
6	1.131621	-1.317623	-0.009626
1	-0.600202	-2.584143	-0.00326
1	1.824955	-2.151596	-0.017025
8	-1.499318	1.885996	-0.000556
6	-2.657831	-0.78516	0.007588
1	-3.154518	-0.367984	-0.877437
1	-3.15016	-0.364656	0.893404
1	-2.858816	-1.857449	0.010023
1	-2.419093	1.603567	-0.000221
1	1.029991	2.07421	-0.015792
6	3.101717	0.276112	0.011903
1	3.444337	0.493511	1.029482
1	3.352911	1.140816	-0.607486

1	3.674599	-0.579595	-0.351872

Phenol, 2,5-dimethyl Cation

Atomic	Coordinates (Angstroms)		
Number	X	Y	Z
6	-0.653766	0.814995	-0.000024
6	0.736841	1.06869	-0.000049
6	-1.200151	-0.534266	0.000013
6	1.624384	0.021468	-0.000034
6	-0.285166	-1.572262	0.00001
6	1.088152	-1.317276	-0.000019
1	-0.637715	-2.596562	0.000022
1	1.780753	-2.152328	-0.000033
8	-1.438239	1.871661	-0.000029
6	-2.680233	-0.748394	0.000041
1	-3.151794	-0.306685	-0.886882
1	-3.151773	-0.30661	0.886938
1	-2.920044	-1.810602	0.000088
1	-2.382949	1.650811	0.000001
1	1.064361	2.101037	-0.000086
6	3.106285	0.216743	0.000036
1	3.553933	-0.260025	0.878864
1	3.378745	1.271133	-0.000827
1	3.554321	-0.26164	-0.877695

Phenol, 2,6-dimethyl

Atomic	Coordinates (Angstroms)		
Number	X	Y	Z
6	0.001154	-0.654146	-0.000009
6	-1.234928	0.008425	-0.000002
6	1.219084	0.040972	-0.000014
6	-1.226949	1.403334	0.000015
6	1.178888	1.437956	-0.000021
6	-0.032531	2.121422	0.000002
1	-2.174907	1.931365	0.000026
1	2.113174	1.989886	-0.000039
1	-0.047179	3.204998	0.000003
8	-0.04601	-2.028332	0.000046
6	2.530011	-0.708101	0.000029
6	-2.515339	-0.785198	0.000002
1	2.641131	-1.346537	-0.885411
1	2.641415	-1.345893	0.885888
1	3.370996	-0.013407	-0.000393
1	-2.57872	-1.436359	-0.876848
1	-3.38166	-0.121249	-0.000108
1	-2.578828	-1.436194	0.876968
1	0.846313	-2.387944	-0.00046

Phenol, 2,6-dimethyl Cation

Atomic	Coordinates (Angstroms)		
Number	X	Y	Z
6	0.013167	-0.654064	-0.000004
6	-1.279796	0.004837	0.000006
6	1.266759	0.058479	-0.000015
6	-1.272594	1.383813	0.00001
6	1.193516	1.42889	-0.000011
6	-0.060567	2.092969	0.000003
1	-2.210217	1.925759	0.000018
1	2.102689	2.018087	-0.000021
1	-0.07901	3.176977	0.000006
8	-0.029147	-1.967828	0.000005
6	2.562422	-0.700271	-0.000014
6	-2.527295	-0.814546	0.000016
1	2.661466	-1.335131	-0.888567
1	2.661595	-1.33488	0.888705
1	3.409085	-0.015183	-0.000177
1	-2.564156	-1.470037	-0.876097
1	-3.409699	-0.176809	-0.000016
1	-2.564179	-1.469973	0.876176
1	0.851934	-2.376832	-0.000007

Phenol, 3,4-dimethyl

Atomic	Coordinates (Angstroms)

Number	X	Y	Z
6	1.648048	0.080977	-0.000002
6	1.272174	-1.258708	0
6	0.669364	1.074488	-0.000005
6	-0.083617	-1.584521	0.000002
6	-0.686312	0.748939	-0.000005
6	-1.078693	-0.607016	0
1	0.986304	2.111351	-0.000008
1	2.024056	-2.042475	-0.000001
8	2.958372	0.488145	-0.000007
6	-1.718655	1.850061	0.000001
1	-1.245666	2.833367	-0.000072
1	-2.368916	1.790051	0.879016
1	-2.369019	1.789968	-0.87893
1	3.535213	-0.282393	0.000038
1	-0.370113	-2.631321	0.000006
6	-2.536552	-0.998659	0.000006
1	-2.648258	-2.084643	0.000018
1	-3.062561	-0.611224	-0.879263
1	-3.062557	-0.611205	0.879269

Phenol, 3,4-dimethyl Cation

Atomic	Coordinates (Angstroms)		
Number	X	Y	Z

6	1.639599	0.108263	-0.000002
6	1.29327	-1.278204	0.000002
6	0.637485	1.113998	-0.000005
6	-0.029357	-1.61852	0.000004
6	-0.69269	0.776816	-0.000003
6	-1.054075	-0.626678	0.000002
1	0.961375	2.147656	-0.00001
1	2.07143	-2.033955	0.000004
8	2.88802	0.525842	-0.000004
6	-1.754045	1.835909	0
1	-1.315092	2.832637	-0.000052
1	-2.39896	1.746712	0.879489
1	-2.399036	1.746651	-0.879426
1	3.535035	-0.197586	-0.000002
1	-0.316979	-2.662916	0.000007
6	-2.480507	-1.043201	0.000004
1	-2.592786	-2.125992	0.000027
1	-3.00361	-0.635139	-0.87386
1	-3.003621	-0.635099	0.873843

Phenol, 3,5-dimethy

Atomic	Coordinates (Angstroms)		
Number	X	Y	Z
6	-0.11596	1.415505	0.000318

6	-1.261044	0.623236	0.011371
6	1.150998	0.83029	-0.013082
6	-1.150941	-0.772254	0.006827
6	1.278187	-0.556782	-0.012446
6	0.12003	-1.346643	-0.004591
1	0.214092	-2.428036	-0.008712
1	2.023166	1.473897	-0.023072
1	-2.242791	1.090601	0.01975
8	-0.170622	2.785545	0.000328
6	2.64388	-1.200986	0.008911
1	3.408603	-0.529224	-0.386615
1	2.659271	-2.120197	-0.581882
1	2.934507	-1.465523	1.031503
6	-2.394303	-1.629405	-0.003048
1	-2.150393	-2.682832	0.14653
1	-2.925634	-1.542959	-0.956653
1	-3.091186	-1.329627	0.785097
1	-1.089735	3.071772	0.005873

Phenol, 3,5-dimethyl Cation

Atomic	Coordinates (Angstroms)		
Number	X	Y	Z
6	0.094163	1.423455	-0.000015
6	1.293556	0.65753	-0.000012

6	-1.19765	0.810229	-0.000015
6	1.214442	-0.713102	0.000006
6	-1.301216	-0.559628	-0.000001
6	-0.090475	-1.311651	0.000019
1	-0.154278	-2.395824	0.00005
1	-2.065804	1.45812	-0.000025
1	2.252977	1.164221	-0.000026
8	0.096229	2.738302	-0.000015
6	-2.62285	-1.270708	0.000006
1	-3.456367	-0.569301	-0.000178
1	-2.713298	-1.913943	0.880455
1	-2.713155	-1.914245	-0.880234
6	2.425724	-1.594044	0.000015
1	2.424132	-2.246547	-0.879109
1	2.424196	-2.24643	0.879228
1	3.349204	-1.016258	-0.000053
1	0.988398	3.121307	-0.000014

A1

Atomic	Coordinates (Angstroms)		
Number	X	Y	Z
6	-2.07981	-1.02989	0
6	-0.78095	-1.48198	-1E-06
6	0.303999	-0.55958	-1E-06

6	0.019853	0.840447	-1E-06
6	-1.29175	1.286952	0.000001
6	-2.32652	0.353248	0.000002
1	-2.90622	-1.72822	0
1	-0.5653	-2.54442	-2E-06
1	-1.51023	2.349131	0.000001
1	-3.34989	0.711011	0.000003
8	1.093942	1.665201	-4E-06
1	0.856597	2.603263	0.000008
6	1.612713	-1.06991	0
1	1.77464	-2.14537	-2E-06
8	2.70799	-0.39679	0.000002
1	2.539719	0.571593	0.000006

A2‡

Atomic	Coordinates (Angstroms)		
Number	X	Y	Z
6	-1.83074	-1.3016	0.022173
6	-0.46829	-1.49252	-0.15817
6	0.377738	-0.36896	-0.17929
6	-0.14089	0.932831	-0.05593
6	-1.51026	1.105145	0.119098
6	-2.34309	-0.00917	0.156676
1	-2.49075	-2.15891	0.052909

1	-0.06314	-2.48983	-0.28206
1	-1.91964	2.104159	0.224579
1	-3.40832	0.134182	0.291693
8	0.779706	1.930783	-0.1157
1	0.375125	2.806836	-0.0782
6	1.806178	-0.49941	-0.42603
1	2.249446	-0.51637	-1.42493
8	2.673947	-0.61674	0.485516
1	2.284192	-0.59026	1.3863

A3

Atomic	Coordinates (Angstroms)		
Number	X	Y	Z
6	1.320894	-1.69835	0.000089
6	-0.02213	-1.43634	0.000019
6	-0.50392	-0.09125	-1E-06
6	0.447612	0.985049	0.00003
6	1.814974	0.696695	0.000094
6	2.236819	-0.62126	0.000122
1	1.685276	-2.71709	0.000112
1	-0.7182	-2.26893	-1.5E-05
1	2.537523	1.50532	0.000116
1	3.300373	-0.83175	0.000165
8	-0.03729	2.226464	-2E-06

1	0.659976	2.896608	0.000038
6	-1.86189	0.238139	-0.00012
1	-2.17594	1.277088	-0.00016
8	-2.8695	-0.57421	-0.00018
1	-2.62882	-1.51545	-0.00015

A4‡

Atomic	Coordinates (Angstroms)		
Number	X	Y	Z
6	-1.02508	-1.78018	0.060869
6	0.281031	-1.31244	0.310535
6	0.529093	0.113361	0.292539
6	-0.50128	1.02451	0.10303
6	-1.79612	0.505462	-0.16019
6	-2.04331	-0.85975	-0.20732
1	-1.25458	-2.83429	0.163244
1	0.831997	-1.91591	1.048056
1	-2.6018	1.199463	-0.38182
1	-3.04668	-1.20774	-0.4206
8	-0.20493	2.327723	0.069006
1	-0.96862	2.883615	-0.1356
6	1.956021	0.353221	0.061368
1	2.471407	1.28754	0.295012
8	2.555459	-0.60396	-0.43507

1	1.36185	-1.46789	-0.40481

A5

Atomic	Coordinates (Angstroms)		
Number	X	Y	Z
6	-1.40882	-1.62056	-2.5E-05
6	0.034697	-1.41888	0.000177
6	0.549564	-0.04264	0.00009
6	-0.33873	1.023388	0.000048
6	-1.73233	0.752539	-9.4E-05
6	-2.26331	-0.54254	-0.00013
1	-1.7904	-2.63587	-2E-06
1	0.488289	-1.98465	0.838886
1	-2.42102	1.594502	-0.00019
1	-3.33772	-0.67792	-0.00025
8	0.153015	2.26965	0.00006
1	-0.52956	2.953087	-0.00004
6	2.038195	0.151695	0.000041
1	2.403129	1.188403	0.000281
8	2.774832	-0.80346	-0.00026
1	0.488949	-1.98516	-0.83772

A6‡

Atomic	Coordinates (Angstroms)		

Number	X	Y	Z
6	-1.36041	-1.66066	-0.03804
6	0.029215	-1.42278	-0.01939
6	0.539634	-0.0574	-0.0123
6	-0.39375	1.018346	0.008716
6	-1.76144	0.736626	0.000084
6	-2.23212	-0.5797	-0.02364
1	-1.72786	-2.67822	-0.07108
1	0.3033	-0.87723	1.079722
1	-2.47311	1.556248	0.018027
1	-3.30188	-0.75215	-0.03468
8	0.129837	2.245404	0.025357
1	-0.53952	2.94347	0.00597
6	2.046552	0.149198	-0.10015
1	2.374395	1.176139	-0.31464
8	2.794941	-0.77691	0.043141
1	0.760395	-2.21797	-0.12302

A7

Atomic	Coordinates (Angstroms)		
Number	X	Y	Z
6	-1.19467	-1.72164	0.063595
6	0.058332	-1.41512	0.484016
6	0.532789	-0.02029	0.527884

6	-0.44913	1.006377	0.134499
6	-1.7168	0.659489	-0.32352
6	-2.0733	-0.67984	-0.34722
1	-1.5323	-2.74923	0.022593
1	0.910684	0.222352	1.535728
1	-2.4187	1.424438	-0.63705
1	-3.0655	-0.94404	-0.69844
8	0.0002	2.24166	0.227815
1	-0.64398	2.909209	-0.05718
6	1.926267	0.170378	-0.29033
1	2.090695	1.213068	-0.61033
8	2.673929	-0.72733	-0.45479
1	0.765122	-2.18655	0.766948

A8‡

Atomic	Coordinates (Angstroms)		
Number	X	Y	Z
6	0.096477	0.900568	0.320564
6	-0.01138	-0.31457	1.016114
6	0.953796	-1.30993	0.791743
6	1.986716	-1.08266	-0.10443
6	2.072441	0.14052	-0.78562
6	1.119052	1.134956	-0.59529
1	-0.73033	-0.40909	1.824187

1	2.886665	0.314489	-1.47883
1	1.179901	2.073489	-1.13576
8	-0.95891	1.779848	0.517225
1	-0.78974	2.660858	0.151932
1	2.739087	-1.84334	-0.27094
6	-2.1757	-0.34118	-0.3555
1	-2.03217	0.763182	-0.04912
8	-2.59101	-1.29054	-0.78099
1	0.897539	-2.24035	1.34323

A9

Atomic	Coordinates (Angstroms)		
Number	X	Y	Z
6	0.320979	-0.25879	0.537225
6	1.001639	-1.28416	-0.08509
6	2.212539	-0.95071	-0.69426
6	2.682541	0.361364	-0.65173
6	1.954102	1.359702	-0.00457
6	0.737767	1.056479	0.608614
1	0.615505	-2.29539	-0.09168
1	2.325365	2.37638	0.026604
1	0.15811	1.819413	1.115107
8	-0.96713	-0.63301	1.17091
1	-1.01395	-0.45819	2.126713

1	3.625158	0.608558	-1.12436
6	-3.2307	0.09817	-0.30092
1	-1.80375	-0.33758	0.673547
8	-4.12843	0.347538	-0.91927
1	2.784819	-1.72176	-1.19469

A10

Atomic	Coordinates (Angstroms)		
Number	X	Y	Z
6	-0.19895	1.224601	-1E-06
6	-0.80092	-0.01608	0.000001
6	-0.16831	-1.23865	0.000002
6	1.22802	-1.20282	0.000001
6	1.900379	0.01875	-1E-06
6	1.197228	1.223967	-1E-06
1	-0.7628	2.15002	-2E-06
1	1.780705	-2.13391	0.000002
1	1.726308	2.16866	-2E-06
8	-2.3087	-0.07858	0.000003
1	-2.74073	0.260612	0.805873
1	-2.74073	0.260526	-0.8059
1	2.983309	0.033172	-1E-06
1	-0.72116	-2.16907	0.000005

A11‡

Atomic	Coordinates (Angstroms)		
Number	X	Y	Z
6	-2.33297	0.141391	-0.17615
6	-1.93285	-1.16194	-0.05929
6	-0.51811	-1.48362	0.151028
6	0.443904	-0.3718	0.211623
6	0.004914	0.936901	0.085265
6	-1.3771	1.169828	-0.09454
1	-3.37409	0.397291	-0.32821
1	-2.64248	-1.97895	-0.12604
1	-0.41634	-2.11302	1.057456
1	-1.7199	2.198761	-0.17609
6	1.92177	-0.69621	0.32601
8	2.617511	-0.5717	-0.64171
1	2.290401	-1.07294	1.297544
1	-0.19816	-2.20049	-0.6315
8	0.914881	1.917131	0.16795
1	0.544072	2.79856	0.033233

A12

Atomic	Coordinates (Angstroms)		
Number	X	Y	Z
6	2.344207	0.33371	0.000039

6	2.069784	-1.01121	-0.00011
6	0.686045	-1.47538	-2.6E-05
6	-0.403	-0.4812	-2.9E-05
6	-0.08729	0.877119	-1.3E-05
6	1.284073	1.249039	0.000091
1	3.363595	0.699319	0.000076
1	2.865321	-1.74817	-0.00022
1	0.548252	-2.17568	-0.84904
1	1.521801	2.310649	0.000202
6	-1.80461	-1.01555	0.000061
8	-2.7881	-0.32619	0.000025
1	-1.86571	-2.12185	0.000088
1	0.548488	-2.17513	0.849529
8	-1.06036	1.783663	-0.00016
1	-0.72942	2.691913	0.000334

A13‡

Atomic	Coordinates (Angstroms)		
Number	X	Y	Z
6	2.335778	0.333803	-0.13838
6	2.059865	-1.00655	-0.06856
6	0.680991	-1.47704	0.085696
6	-0.39801	-0.47023	0.113066
6	-0.08449	0.876186	0.060494

6	1.27772	1.254222	-0.05125
1	3.352476	0.693047	-0.23925
1	2.851951	-1.74588	-0.1214
1	0.472351	-2.24704	-0.68124
1	1.497232	2.318546	-0.05804
6	-1.82135	-0.96558	0.168363
8	-2.70462	-0.39686	-0.4119
1	-1.98495	-1.90735	0.72514
1	0.61975	-2.09037	1.00893
8	-1.02735	1.819149	0.2701
1	-1.25617	2.331892	-0.51639

A14

Atomic	Coordinates (Angstroms)		
Number	X	Y	Z
6	-2.31892	0.454498	0.000033
6	-2.12278	-0.90923	-1.4E-05
6	-0.77539	-1.47328	-4.6E-05
6	0.366525	-0.53739	0.000005
6	0.135207	0.836819	-4.9E-05
6	-1.2137	1.306837	0.000062
1	-3.31784	0.873079	0.000083
1	-2.96548	-1.5925	-0.00008
1	-0.6982	-2.17752	0.852586

1	-1.35316	2.384255	0.000189
6	1.750727	-1.05005	0.000079
8	2.707006	-0.29597	-3.2E-05
1	1.890304	-2.14347	0.000415
1	-0.69826	-2.1772	-0.85297
8	1.07449	1.762734	-5.1E-05
1	1.960592	1.329992	0.000017

A15‡

Atomic	Coordinates (Angstroms)		
Number	X	Y	Z
6	-2.31245	0.177193	-0.16797
6	-1.91777	-1.16579	-0.01239
6	-0.56809	-1.44849	0.185836
6	0.384477	-0.37988	0.187887
6	-0.01505	0.959395	0.117367
6	-1.39893	1.210906	-0.08287
1	-3.35705	0.408937	-0.33886
1	-2.65909	-1.9533	0.039914
1	-0.36675	-2.35865	0.769045
1	-1.70144	2.240277	-0.23829
6	1.731521	-0.82418	-0.06566
8	2.809181	-0.37836	-0.15569
1	1.541887	-2.09577	-0.39133

8	0.796185	2.007589	0.092022
1	1.731509	1.783372	0.212128
1	0.545762	-2.2336	-0.51652

A16

Atomic	Coordinates (Angstroms)		
Number	X	Y	Z
6	2.28068	-0.09512	-6.7E-05
6	1.707734	-1.38727	0.000005
6	0.348719	-1.5261	0.000066
6	-0.47037	-0.34175	0.000069
6	0.115971	0.970836	0.000028
6	1.509554	1.053414	-0.00003
1	3.360329	0.002316	-0.00011
1	2.341281	-2.2647	-1.3E-05
1	-0.12456	-2.49985	0.000164
1	1.958684	2.038758	-0.00006
6	-1.82386	-0.51186	-3.3E-05
8	-2.94719	-0.65561	-7.4E-05
8	-0.55612	2.11799	0.000024
1	-1.51984	2.051573	0.000187

A17

Atomic	Coordinates (Angstroms)		

Number	X	Y	Z
6	-1.11946	1.15975	-0.01738
6	-1.8963	0.00764	0.041542
6	-1.19994	-1.23466	0.012467
6	0.113645	-1.02821	0.075896
6	1.07284	-0.10827	-0.08501
6	0.272139	1.09213	-0.02595
1	-1.58958	2.136133	-0.02161
1	-2.97613	0.006506	-0.04379
1	0.884605	1.956344	0.238314
8	2.38782	-0.0443	-0.01378
1	2.80282	-0.90056	0.167247
1	-1.68187	-2.17435	-0.23932

A18‡

Atomic	Coordinates (Angstroms)		
Number	X	Y	Z
6	-1.35031	1.07505	-0.10088
6	-1.97474	-0.20944	-0.19013
6	-1.07996	-1.21275	0.084524
6	0.168036	-0.60176	0.379886
6	1.301203	-0.23418	-0.07371
6	-0.02031	0.989003	0.224248
1	-1.87032	2.023662	-0.14731

1	-2.99906	-0.37178	-0.49754
1	0.593195	1.766889	0.659755
8	2.508523	0.049026	-0.27892
1	3.143563	-0.37069	0.336837
1	-1.19906	-2.27582	-0.0641

A19

Atomic	Coordinates (Angstroms)		
Number	X	Y	Z
6	0.259163	-0.00054	-0.0204
6	-0.62668	-1.2089	-0.00412
1	-0.26524	-2.22422	-0.01181
6	-1.88607	-0.73706	0.009603
1	-2.77521	-1.35321	0.010711
6	-0.62573	1.208709	-0.00403
1	-0.26338	2.223707	-0.0118
6	-1.88548	0.737883	0.00949
1	-2.77412	1.354771	0.010474
6	1.541954	-0.0004	0.037952
8	2.764159	0.000072	-0.11048
1	3.301725	0.000209	0.715251

Table S3. Optimized structures by G4 method

Toluene

Atomic	Coordinates (Angstroms)		
Number	X	Y	Z
6	-1.900961	-0.000043	0.007764
6	-1.198526	-1.202714	0.001834
6	0.193773	-1.199731	-0.008371
6	0.911346	0.000063	-0.010272
6	0.193698	1.199773	-0.008358
6	-1.198633	1.202657	0.001845
1	-2.985862	-0.000092	0.01249
1	-1.734944	-2.146265	0.000805
1	0.733484	-2.142531	-0.016979
1	0.733339	2.142608	-0.016949
1	-1.735111	2.146173	0.000824
6	2.420778	0.000028	0.008866
1	2.80534	-0.003607	1.036454
1	2.827474	-0.883838	-0.491328
1	2.827424	0.887352	-0.485165

Toluene Cation

Atomic	Coordinates (Angstroms)		
Number	X	Y	Z
6	1.880672	-0.000007	0.006579
6	1.181434	1.238701	0.001367
6	-0.186339	1.239939	-0.007532

6	-0.914838	0.000011	-0.015578
6	-0.186346	-1.23993	-0.007532
6	1.181426	-1.238707	0.001367
1	2.966218	-0.00001	0.016197
1	1.739965	2.167467	0.003464
1	-0.743143	2.170564	-0.012265
1	-0.743161	-2.170549	-0.012264
1	1.739947	-2.167479	0.003464
6	-2.390739	0.000004	-0.002358
1	-2.724436	-0.00021	1.053362
1	-2.813519	0.900615	-0.454764
1	-2.813494	-0.900466	-0.455079

Anisole

Atomic	Coordinates (Angstroms)		
Number	X	Y	Z
6	-0.454938	-0.271763	-0.000007
6	0.494969	-1.30041	-0.000002
6	-0.033795	1.060119	-0.00001
6	1.848229	-0.997375	0.000001
6	2.278222	0.331116	-0.000001
6	1.332129	1.348555	-0.000007
1	0.142746	-2.325652	0.000001
1	2.573888	-1.804342	0.000006

1	1.649867	2.386374	-0.00001
1	3.336757	0.565419	0.000001
1	-0.750651	1.87082	-0.000017
8	-1.756647	-0.671189	-0.000013
6	-2.761114	0.323822	0.000027
1	-2.703412	0.959649	0.893111
1	-3.714769	-0.206808	0.000052
1	-2.70347	0.959665	-0.893049

Anisole Cation

Atomic	Coordinates (Angstroms)		
Number	X	Y	Z
6	-0.451862	-0.254722	0.000002
6	0.511935	-1.322413	-0.000002
6	-0.03136	1.11138	-0.000002
6	1.849158	-1.024421	-0.000009
6	2.261552	0.324712	-0.000012
6	1.311949	1.380994	-0.000009
1	0.13662	-2.338952	0.000001
1	2.588254	-1.816535	-0.000011
1	1.65999	2.407442	-0.000013
1	3.319551	0.563786	-0.000017
1	-0.759737	1.911161	-0.000002
8	-1.694344	-0.647057	0.000008

6	-2.809292	0.28402	0.000019
1	-2.772962	0.898599	0.901934
1	-3.696445	-0.344928	0.000042
1	-2.772995	0.898577	-0.901913

p-Methyphenol

Atomic	Coordinates (Angstroms)		
Number	X	Y	Z
6	-1.440197	-0.018479	0.001147
6	-0.749849	1.191598	-0.000578
6	-0.725262	-1.217973	-0.000455
6	0.643778	1.196831	-0.003142
6	0.662269	-1.193365	-0.003035
6	1.377273	0.010862	-0.003332
1	1.166648	2.148795	-0.005537
1	-1.298342	2.130274	-0.001691
8	-2.803471	-0.094174	0.002497
1	-3.16568	0.79775	0.002783
1	1.20501	-2.134603	-0.005608
1	-1.27264	-2.153691	-0.001338
6	2.886741	0.016687	0.004262
1	3.287548	-0.390214	0.940415
1	3.280547	1.030511	-0.108817
1	3.296159	-0.592397	-0.809383

p-Methyphenol Cation

Atomic	Coordinates (Angstroms)		
Number	X	Y	Z
6	1.433384	0.008152	0.000038
6	0.732465	-1.234636	-0.000045
6	0.724248	1.249892	-0.000033
6	-0.632475	-1.221786	-0.000176
6	-0.642718	1.237401	-0.000172
6	-1.36466	0.010241	-0.000239
1	-1.18353	-2.156131	-0.00027
1	1.288451	-2.166959	-0.000044
8	2.742218	0.088828	0.000159
1	3.177841	-0.779067	0.000188
1	-1.193655	2.171045	-0.000268
1	1.299717	2.16824	-0.00001
6	-2.849932	-0.015061	0.000123
1	-3.219985	-0.563378	0.877966
1	-3.220923	-0.572749	-0.871238
1	-3.287529	0.98315	-0.00457

m-Methyphenol

Atomic	Coordinates (Angstroms)		
Number	X	Y	Z

6	1.216737	-0.368868	0.000055
6	1.315835	1.021251	0.00003
6	-0.037794	-0.98237	-0.000014
6	0.151303	1.786304	-0.00007
6	-1.202667	-0.219087	-0.000092
6	-1.097526	1.177953	-0.000139
1	-0.082478	-2.066225	-0.000013
1	-1.997038	1.785298	-0.000242
1	0.226147	2.869014	-0.000121
1	2.291718	1.49947	0.000059
6	-2.558383	-0.883467	0.000038
1	-2.470984	-1.972869	-0.001985
1	-3.14339	-0.59152	-0.879355
1	-3.141622	-0.59473	0.881684
8	2.310896	-1.184704	0.000122
1	3.105448	-0.641101	0.000144

m-Methyphenol Cation

Atomic	Coordinates (Angstroms)		
Number	X	Y	Z
6	-1.205841	-0.408505	-0.000071
6	-1.365962	1.021666	0.000067
6	0.081971	-1.018019	-0.000065
6	-0.238771	1.804315	0.000064

6	1.212046	-0.232479	-0.000036
6	1.039684	1.195604	-0.000117
1	0.131583	-2.101568	-0.000075
1	1.924025	1.82689	-0.000406
1	-0.31944	2.885777	0.000133
1	-2.362134	1.455261	0.000177
6	2.596447	-0.807549	0.000111
1	2.582578	-1.898112	0.002175
1	3.154108	-0.464694	0.879423
1	3.15297	-0.468018	-0.881219
8	-2.235801	-1.225001	-0.000005
1	-3.094723	-0.76572	0.000118

o-Methyphenol

Atomic	Coordinates (Angstroms)		
Number	X	Y	Z
6	-0.411081	0.752961	0.000021
6	0.863931	1.314473	0.000001
6	-0.588297	-0.639584	-0.000006
6	1.988324	0.492285	-0.000025
6	0.554082	-1.437558	-0.000045
6	1.83635	-0.889465	-0.000049
1	2.705308	-1.537878	-0.000071
1	2.977484	0.937782	-0.000031

1	0.973996	2.396241	-0.000001
8	-1.544467	1.518069	0.000028
1	-1.294098	2.447547	0.000347
1	0.431446	-2.516859	-0.000067
6	-1.977149	-1.221298	0.000021
1	-2.546603	-0.89425	-0.876922
1	-2.546477	-0.89445	0.877122
1	-1.942269	-2.31358	-0.000104

o-Methyphenol Cation

Atomic	Coordinates (Angstroms)		
Number	X	Y	Z
6	-0.357487	0.792241	0.000035
6	0.972877	1.299829	0.000024
6	-0.645277	-0.636681	-0.000013
6	2.014538	0.410849	-0.000032
6	0.446939	-1.494827	-0.000069
6	1.75439	-0.996702	-0.000077
1	2.592394	-1.686684	-0.000118
1	3.039739	0.765098	-0.000042
1	1.141483	2.372995	0.000058
8	-1.413505	1.577078	0.000087
1	-1.186015	2.524242	0.000116
1	0.280111	-2.566596	-0.000108

6	-2.061007	-1.105521	0.000015
1	-2.598433	-0.723063	-0.87627
1	-2.598289	-0.723338	0.876512
1	-2.112787	-2.194409	-0.000142

Phenol, 2,3-dimethyl

Atomic	Coordinates (Angstroms)		
Number	X	Y	Z
6	-1.226828	-0.028045	-0.000013
6	-1.327251	1.36191	-0.000031
6	0.01979	-0.673471	-0.000039
6	-0.168936	2.124655	-0.000008
6	1.186341	0.112266	-0.000011
6	1.077054	1.504742	0.000008
8	-2.401603	-0.726466	0.000121
6	0.052126	-2.184924	-0.000017
1	-0.44734	-2.604473	-0.884603
1	-0.44578	-2.60442	0.885462
1	1.070229	-2.573346	-0.000976
1	-2.213387	-1.66957	-0.000357
1	-2.312393	1.813533	-0.000039
1	-0.236817	3.207582	-0.000007
1	1.980048	2.106371	0.000034
6	2.560288	-0.519329	0.000019

1	3.335931	0.250244	0.000143
1	2.723473	-1.150414	-0.881285
1	2.723349	-1.150595	0.881213

Phenol, 2,3-dimethyl Cation

Atomic	Coordinates (Angstroms)		
Number	X	Y	Z
6	-1.21295	-0.161822	-0.000021
6	-1.491167	1.244151	-0.000023
6	0.122064	-0.699388	-0.000053
6	-0.438576	2.111215	0
6	1.174963	0.202058	0.000013
6	0.880388	1.596306	0.000028
8	-2.27184	-0.935584	0.000032
6	0.291325	-2.19279	-0.000069
1	-0.165221	-2.649814	-0.888354
1	-0.163729	-2.649615	0.889086
1	1.34117	-2.479999	-0.00095
1	-2.047768	-1.880715	0.000049
1	-2.527224	1.560459	-0.000027
1	-0.597715	3.182757	0.000006
1	1.711548	2.294299	0.000056
6	2.614902	-0.237366	0.000068
1	3.28838	0.620727	0.00024

1	2.844867	-0.84367	-0.882298
1	2.844724	-0.843931	0.882287

Phenol, 2,4-dimethyl

Atomic	Coordinates (Angstroms)		
Number	X	Y	Z
6	-1.112653	-0.639211	0.003434
6	-0.125757	-1.622885	-0.001865
6	-0.761232	0.717824	-0.000347
6	1.215184	-1.262836	-0.008758
6	0.596017	1.045068	-0.007052
6	1.603493	0.079908	-0.008578
1	0.872095	2.09674	-0.012561
8	-2.414093	-1.058648	0.007135
6	-1.82958	1.783523	-0.001901
1	-2.474902	1.723909	0.885329
1	-2.480483	1.71612	-0.884479
1	-1.384402	2.781062	-0.007573
1	-2.995017	-0.292007	0.009315
1	-0.430442	-2.663402	-0.004625
1	1.97308	-2.040937	-0.015921
6	3.061735	0.470266	0.011078
1	3.203074	1.502262	-0.322861
1	3.658794	-0.177358	-0.639114

1	3.487709	0.392862	1.01935

Phenol, 2,4-dimethyl Cation

Atomic	Coordinates (Angstroms)		
Number	X	Y	Z
6	1.089471	-0.661397	0.000013
6	0.069341	-1.656834	-0.000003
6	0.778542	0.747814	-0.000037
6	-1.236472	-1.268079	-0.000027
6	-0.553151	1.091185	-0.000059
6	-1.586351	0.120828	-0.00004
1	-0.828807	2.140294	-0.000103
8	2.326009	-1.103658	0.000082
6	1.887048	1.758118	-0.000014
1	2.525233	1.657407	-0.887809
1	2.524546	1.658037	0.888347
1	1.488254	2.773201	-0.000544
1	2.981676	-0.388056	0.000094
1	0.371115	-2.697461	0.000009
1	-2.027807	-2.00964	-0.000037
6	-3.019327	0.519126	0.000018
1	-3.155413	1.600676	-0.000622
1	-3.530496	0.095718	0.875515
1	-3.530983	0.094539	-0.874604

Phenol, 2,5-dimethyl

Atomic	Coordinates (Angstroms)		
Number	X	Y	Z
6	-0.673256	0.79194	-0.000932
6	0.696415	1.041821	-0.008228
6	-1.16884	-0.521354	0.00304
6	1.616751	-0.006353	-0.009724
6	-0.237907	-1.559412	-0.001639
6	1.13194	-1.316226	-0.009136
1	-0.599687	-2.583843	-0.00334
1	1.827699	-2.149086	-0.015734
8	-1.500952	1.879536	-0.001368
6	-2.654633	-0.782093	0.007031
1	-3.152875	-0.36167	-0.877533
1	-3.148392	-0.358127	0.892368
1	-2.860118	-1.855071	0.009647
1	-2.415845	1.582315	0.000147
1	1.02815	2.075059	-0.014434
6	3.099135	0.277223	0.011562
1	3.447378	0.496013	1.028571
1	3.350347	1.143915	-0.607606
1	3.673328	-0.579064	-0.352991

Phenol, 2,5-dimethyl Cation

Atomic	Coordinates (Angstroms)		
Number	X	Y	Z
6	-0.654309	0.813767	-0.000026
6	0.736717	1.068568	-0.000049
6	-1.199292	-0.534496	0.000013
6	1.624433	0.022624	-0.000032
6	-0.284895	-1.571273	0.000013
6	1.088532	-1.314821	-0.000015
1	-0.637225	-2.596443	0.000026
1	1.783033	-2.149387	-0.000027
8	-1.439738	1.866381	-0.000032
6	-2.680249	-0.745034	0.000039
1	-3.151415	-0.30062	-0.887412
1	-3.151394	-0.300549	0.887466
1	-2.92316	-1.808182	0.000085
1	-2.382134	1.635789	-0.000004
1	1.062446	2.102087	-0.000087
6	3.106939	0.217011	0.000035
1	3.55493	-0.261432	0.879347
1	3.380258	1.272655	-0.000821
1	3.555305	-0.263034	-0.878192

Phenol, 2,6-dimethyl

Atomic	Coordinates (Angstroms)

Number	X	Y	Z
6	0.001757	-0.654891	0
6	-1.233248	0.008279	0.000001
6	1.217321	0.042811	-0.000016
6	-1.226522	1.401819	0.000008
6	1.176518	1.438277	-0.00002
6	-0.034108	2.120896	-0.000002
1	-2.176481	1.928153	0.000013
1	2.112176	1.989636	-0.00004
1	-0.049635	3.205127	-0.000002
8	-0.043894	-2.023503	0.000054
6	2.526445	-0.708494	0.000005
6	-2.511667	-0.788104	0.000006
1	2.636859	-1.350362	-0.884983
1	2.637054	-1.349934	0.88527
1	3.37111	-0.015861	-0.00029
1	-2.574789	-1.441926	-0.876803
1	-3.38192	-0.127068	-0.000115
1	-2.574908	-1.441742	0.876945
1	0.8527	-2.371559	-0.000313

Phenol, 2,6-dimethyl Cation

Atomic	Coordinates (Angstroms)		
Number	X	Y	Z

6	0.012357	-0.652482	-0.000002
6	-1.279343	0.005491	0.000005
6	1.265683	0.059218	-0.000016
6	-1.271868	1.383374	0.000008
6	1.193823	1.429106	-0.000013
6	-0.059425	2.092683	0.000001
1	-2.21015	1.925687	0.000015
1	2.103938	2.018184	-0.000023
1	-0.077734	3.177505	0.000004
8	-0.028002	-1.964099	0.000013
6	2.558947	-0.703377	-0.000019
6	-2.52595	-0.816571	0.000016
1	2.655292	-1.34064	-0.888927
1	2.655403	-1.340427	0.889029
1	3.409149	-0.020159	-0.000159
1	-2.561389	-1.473378	-0.87679
1	-3.410932	-0.179996	-0.000018
1	-2.561412	-1.473313	0.876871
1	0.856501	-2.365319	0.000012

Phenol, 3,4-dimethyl

Atomic	Coordinates (Angstroms)		
Number	X	Y	Z
6	1.649544	0.08392	-0.000002

6	1.273271	-1.255761	0
6	0.667736	1.075125	-0.000004
6	-0.080544	-1.582821	0.000002
6	-0.685552	0.747577	-0.000004
6	-1.075692	-0.607448	0
1	0.983444	2.11307	-0.000007
1	2.02758	-2.038236	-0.000001
8	2.953928	0.489249	-0.000007
6	-1.720728	1.845495	0.000001
1	-1.251027	2.832002	-0.00007
1	-2.373698	1.784319	0.878835
1	-2.373798	1.784237	-0.878753
1	3.521097	-0.288556	0.000026
1	-0.367843	-2.630363	0.000005
6	-2.532695	-0.999481	0.000006
1	-2.645086	-2.08687	0.000018
1	-3.062068	-0.611626	-0.879031
1	-3.062066	-0.611605	0.879035

Phenol, 3,4-dimethyl Cation

Atomic	Coordinates (Angstroms)		
Number	X	Y	Z
6	1.640636	0.110841	-0.000002
6	1.295526	-1.275886	0.000003

6	0.636168	1.114914	-0.000006
6	-0.026769	-1.617363	0.000006
6	-0.692806	0.775857	-0.000004
6	-1.051629	-0.627106	0.000003
1	0.959516	2.14944	-0.000012
1	2.076464	-2.029671	0.000005
8	2.886489	0.527033	-0.000006
6	-1.75778	1.831913	-0.000001
1	-1.32195	2.831575	-0.000043
1	-2.404253	1.739731	0.879795
1	-2.404313	1.739682	-0.879747
1	3.528543	-0.200637	-0.000005
1	-0.315486	-2.662321	0.00001
6	-2.478792	-1.04419	0.000006
1	-2.591642	-2.12832	0.000029
1	-3.003051	-0.634837	-0.874159
1	-3.003062	-0.634797	0.874145

Phenol, 3,5-dimethy

Atomic	Coordinates (Angstroms)		
Number	X	Y	Z
6	-0.12257	1.417162	0.000066
6	-1.262047	0.617443	0.010519
6	1.146144	0.835177	-0.012209

6	-1.145703	-0.77598	0.006346
6	1.279464	-0.550104	-0.011287
6	0.126409	-1.344346	-0.004228
1	0.225456	-2.426045	-0.008229
1	2.016021	1.482972	-0.021476
1	-2.246873	1.080102	0.018057
8	-0.184648	2.780999	-0.000199
6	2.647719	-1.188221	0.008956
1	3.410551	-0.512467	-0.387347
1	2.666996	-2.109064	-0.581816
1	2.944848	-1.454097	1.030939
6	-2.384393	-1.639164	-0.003162
1	-2.135665	-2.692865	0.146633
1	-2.920331	-1.556652	-0.956263
1	-3.085136	-1.3423	0.784711
1	-1.108824	3.050627	0.006371

Phenol, 3,5-dimethyl Cation

Atomic	Coordinates (Angstroms)		
Number	X	Y	Z
6	0.09604	1.42413	-0.000017
6	1.293618	0.655289	-0.000011
6	-1.196475	0.812949	-0.000014
6	1.212504	-0.714388	0.000005

6	-1.302214	-0.555962	0.000001
6	-0.092647	-1.30893	0.000018
1	-0.158443	-2.393866	0.000046
1	-2.063319	1.463522	-0.000023
1	2.254083	1.161382	-0.000024
8	0.102957	2.736275	-0.000018
6	-2.624153	-1.266443	0.000011
1	-3.458462	-0.563818	-0.000172
1	-2.714182	-1.911585	0.880808
1	-2.714041	-1.911888	-0.880577
6	2.421471	-1.599082	0.000015
1	2.417436	-2.253101	-0.879572
1	2.417501	-2.252984	0.87969
1	3.34769	-1.023048	-0.000054
1	0.99922	3.109803	-0.000017

References:

1. Mukai, T.; Nakazawa, T.; Shishido, T., Troponoid-benzenoid rearrangement by pyrolytic process. *Tetrahedron Lett.* **1967,** *8*, 2465-2469.
2. Dits, H.; Nibbering, N. M. M.; Verhoeven, J. W., A MINDO/3- and ion cyclotron resonance study of some $C_7H_7O^+$ ions. *Chem. Phys. Lett.* **1977,** *51*, 95-98.
3. Dewar, M. J. S.; Landman, D., Gaseous ions. 3. MINDO/3 calculations for the rearrangements of substituted benzyl cations. *J. Am. Chem. Soc.* **1977,** *99*, 4633-4639.
4. Cassady, C. J.; Freiser, B. S.; Russell, D. H., Structural determination of $[C_7H_7O]^+$ ions in the gas phase by ion cyclotron resonance spectrometry. *J. Mass Spectrom.* **1983,** *18*, 378-387.
5. Holzmann, G.; Frenking, G.; Steiner, B., Thermal- and electron impact-induced decarbonylation of tropones: a comparison of neutral and radical-cationic pericyclic reaction mechanisms. *J. Chem. Soc., Perkin Trans. 2* **1984**, 1943-1948.

6. Chiavarino, B.; Crestoni, M. E.; Fornarini, S.; Dopfer, O.; Lemaire, J.; Maître, P., IR Spectroscopic Features of Gaseous $C_7H_7O^+$ Ions: Benzylium versus Tropylium Ion Structures. *J. Phys. Chem. A* **2006,** *110*, 9352-9360.
7. Bouchoux, G., Heats of formation and protonation thermochemistry of gaseous benzaldehyde, tropone and quinone methides. *Chem. Phys. Lett.* **2010,** *495*, 192-197.
